# Development and validation of an RBP gene signature for prognosis prediction in colorectal cancer based on WGCNA

**DOI:** 10.1186/s41065-023-00274-z

**Published:** 2023-03-10

**Authors:** Lu Cao, Lili Duan, Rui Zhang, Wanli Yang, Ning Yang, Wenzhe Huang, Xuemin Chen, Nan Wang, Liaoran Niu, Wei Zhou, Junfeng Chen, Yiding Li, Yujie Zhang, Jinqiang Liu, Daiming Fan, Hong Liu

**Affiliations:** 1 Department of Biomedical Engineering, Air Force Hospital of Eastern Theater Command, 210001 Nanjing, Jiangsu Province China; 2 Division of Digestive Surgery, State Key Laboratory of Cancer Biology and National Clinical Research Center for Digestive Diseases, Xijing Hospital of Digestive Diseases, Fourth Military Medical University, 710032 Xi’an, Shaanxi Province China; 3 Department of Biomedical Engineering, Jinling Hospital, Medical School of Nanjing University, 210002 Nanjing, Jiangsu Province China; 4 College of Otolaryngology and Head and Neck Surgery, State Key Lab of Hearing Science, Beijing Key Lab of Hearing Impairment Prevention and Treatment, Chinese PLA General Hospital, National Clinical Research Center for Otolaryngologic Diseases, Ministry of Education, Beijing, China; 5 Department of Hematology, The Fifth Medical Center, Chinese PLA General Hospital, Beijing, China; 6 Department of Histology and Embryology, School of Basic Medicine, Xi’an Medical University, Xi’an, China

**Keywords:** Colorectal cancer, RNA binding protein, Prognostic model, Weighted gene co-expression network analysis (WGCNA)

## Abstract

**Background:**

RNA binding proteins (RBPs) have been implicated in oncogenesis and progression in various cancers. However, the potential value of RBPs as prognostic indicators and therapeutic targets in colorectal cancer (CRC) requires further investigation.

**Methods:**

Four thousand eighty two RBPs were collected from literature. The weighted gene co-expression network analysis (WGCNA) was performed to identify prognosis-related RBP gene modules based on the data attained from the TCGA cohorts. LASSO algorithm was conducted to establish a prognostic risk model, and the validity of the proposed model was confirmed by an independent GEO dataset. Functional enrichment analysis was performed to reveal the potential biological functions and pathways of the signature and to estimate tumor immune infiltration. Potential therapeutic compounds were inferred utilizing CMap database. Expressions of hub genes were further verified through the Human Protein Atlas (HPA) database and RT-qPCR.

**Results:**

One thousand seven hundred thirty four RBPs were differently expressed in CRC samples and 4 gene modules remarkably linked to the prognosis were identified, based on which a 12-gene signature was established for prognosis prediction. Multivariate Cox analysis suggested this signature was an independent predicting factor of overall survival (*P* < 0.001; HR:3.682; CI:2.377–5.705) and ROC curves indicated it has an effective predictive performance (1-year AUC: 0.653; 3-year AUC:0.673; 5-year AUC: 0.777). GSEA indicated that high risk score was correlated with several cancer-related pathways, including cytokine-cytokine receptor cross talk, ECM receptor cross talk, HEDGEHOG signaling cascade and JAK/STAT signaling cascade. ssGSEA analysis exhibited a significant correlation between immune status and the risk signature. Noscapine and clofazimine were screened as potential drugs for CRC patients with high-risk scores. TDRD5 and GPC1 were identified as hub genes and their expression were validated in 15 pairs of surgically resected CRC tissues.

**Conclusion:**

Our research provides a depth insight of RBPs’ role in CRC and the proposed signature are helpful to the personalized treatment and prognostic judgement.

**Supplementary Information:**

The online version contains supplementary material available at 10.1186/s41065-023-00274-z.

## Introduction

Colorectal cancer (CRC) is the third most common malignancies, ranking the second in cancer mortality worldwide [1]. Although great progress has been achieved in diagnostic and therapeutic approaches, CRC patients suffered from poor prognosis.

RNA-binding proteins (RBPs) are a group of intrinsically pleiotropic proteins that interact with their targets RNA via RNA-binding domains (RBDs), forming ribonucleoprotein complexes and further implicating in RNA metabolism and post-transcriptionally gene regulation. RBPs serve as crucial regulators of various cellular processes, including cell transport, development, differentiation, and metabolism. Mounting evidences exhibit that dysregulation of RBPs is critical for tumorigenesis and progression in colorectum. For instance, an RBP CELF1, which is highly expressed in numerous human malignant tumours, promotes cell migration, invasion, and chemoresistance in CRC [2].Another evolutionarily conserved RBP LIN28B could modulate biogenesis of let-7 microRNAs, further promoting CRC growth and progression [3–6]. Previous research demonstrated that RBP RBM3 was upregulated in CRC and overexpression RBM3 enhanced stem-like properties and drug resistance of CRC [7, 8]. Interestingly, some RBPs have a dichotomous role in CRC. For example, IMP1 has a critical role in modulation of cell cycle progress along with migration in CRC cells [9]. IMP1 was highly expressed in most CRC samples [10] and aberrant expression of IMP1 was linked to enhanced metastasis and worse prognosis [11, 12], whereas stromal IMP1 served as a tumour-suppressive factor in colon [13, 14]. Therefore, further investigation on RBPs may provide novel ideas for screening new diagnostic and therapeutic targets of CRC.

In the present study, we thoroughly reviewed another two studies which examined the prognostic significance of RBPs in CRC. Firstly, a 4-gene model (SMAD6, UPF3B, RP9 and NOL3) was constructed by Zheng Z et al. whose 3-year AUC reached 0.645 and 5-year AUC reached 0.672 [15]. Secondly, Xuehui F et al. established a 12-gene model (NOP14, MRPS23, MAK16, TDRD6, POP1, TDRD5, TDRD7, PPARGC1A, LIN28B, CELF4, LRRFIP2 and MSI2), which significantly divided CRC patients into high- and low-risk groups in terms of OS (*P* < 0.001) [16]. Unfortunately, those two studies failed to fully collect potential RBPs from different resources (1542 of Zheng Z’s and 1493 of Xuehui F’s). And lack the analysis of RBDs which are essential for RBPs to perform their functions. In this study, a novel method named weighted gene co-expression network analysis (WGCNA) was used to identify the key prognostic genes in a co-regulated gene network level instead of an individual gene level, which is more compliant with biology laws. And more comprehensive analyses including immune cell infiltration quantification, potential drugs prediction and in vitro experiments validation were also encompassed in this study. The detailed comparisons among these studies were shown in Table S1.

Herein, we utilized WGCNA to identify the prognosis-correlated modules and hub genes. Next, we established a prognostic signature based on 12 RBP genes and validated it in an independent GEO cohort. GO and KEGG analyses were employed to reveal the underlying functional mechanisms of RBPs in CRC. Gene set enrichment analysis (GSEA) was used to explore functions of this signature and single-sample gene set enrichment analysis (ssGSEA) was conducted to reveal its relationship with immune cell infiltration and functions. We also created a nomogram to estimate an individual’s survival chance through the integration of clinical characteristics and the proposed signature. Potential drugs were identified using CMap database. Finally, the mRNA and protein expression levels of hub genes were verified.

## Materials and methods

### Data acquisition

Based the research of Zhixing Wang in 2020, a list of 4082 human RBP genes was comprehensively integrated from six sources: Gerstberger [17], SONAR [18], the Gene Ontology project, Poly(A)-binding protein [19], CARIC [20], and XRNAX [21]. The RNA sequencing (RNA-seq) data and the matching clinical profiles of 476 CRC patients were obtained from TCGA data resource (https://portal.gdc.cancer.gov/), containing 42 non-tumour samples and 488 tumour samples. Meanwhile, a cohort of 122 CRC patients from the GEO data resource (https://www.ncbi.nlm.nih.gov/geo/) (GSE38832) was employed as an independent external test set. This cohort contained 122 tumour samples. The R software (version 4.0.2) and package “limma” were used to normalize and process the data. The current research complies with TCGA and GEO policies and guidelines.

### Expression and domain analysis of RBPs in CRC

Firstly, the differently expressed RBPs between tumour and non-tumour tissue in the TCGA dataset were uncovered with the cut-off of FDR < 0.05 and |logFC|>0.5 using the R “limma” package. Next, we extracted 1394 protein binding domains of the differently expressed RBPs from the Pfam [22] data resource (http://pfam.xfam.org) using an online tool David [23] (V.6.8, https://david.ncifcrf.gov/). RNA binding domains (RBDs) are the sites through which RBPs interreact with their target RNAs. Finally, on the basis of the RBDs information, RBPs were stratified into two families, namely the canonical subfamily with canonical RBDs (experimentally or structurally verified to directly bind RNAs) and the non-canonical subfamily. The list of canonical RBDs was obtained from literature [24]. Enrichment analyses regarding these two subfamilies were conducted.

### Weighted gene co-expression network analysis

WGCNA is a systematic biology method for determining the association patterns among genes across different samples. It can be used to identify highly covarying gene sets (modules) and to identify candidate biomarkers or therapeutic targets based on the association between modules and sample phenotypes [25]. This approach focuses on exploring associations between external traits and co-expression gene sets instead of individual genes, which is more comply with biological laws. It has been widely used in various cancer researches. In this study, the expression pattern of the 1734 differently expressed RBPs and their matching clinical features (age, gender, overall survival time, survival status, and stage) in the TCGA cohort were employed to create a co-expression network using the R “WGCNA” package (V.4.0.2). The WGCNA approach was performed as documented previously [25]. First, to remove outlier samples, a hierarchical clustering analysis of CRC tumour samples on the basis of the expression of RBPs was performed. After that, we screened the estimated soft threshold power (β) to ensure the construction of scale-free networks, which is more in line with the law of biology. Herein, β = 5 (Figure S1 scale free R^2^ = 0.885) was employed. Considering the TOM-based dissimilarity measure, average linkage hierarchical clustering with a min-Module size (gene group) of 20 was carried out. Moreover, RBPs with similar expression modes were categorized into the same modules and similar modules were merged. Next, we calculated the module eigengenes (MEs) and gene significance (GS). MEs exhibit the first principal component-linked module, whose value representing all genes in the module. GS was defined as the association of genes with traits and was employed to quantify the relationship of individual genes with the clinical traits of interest. Based on these two parameters, modules that are remarkably related with the OS time or tumour stage were uncovered as prognosis-related modules. The PPI network of the genes from these prognosis-related modules were constructed using the STRING website and the cut-off confidence was set as 0.9 (https://string-db.org/cgi/input.pl, version 11.0) and Cytoscape software (Version 3.8.2).

### Construction and validation of prognostic models based on RBPs

Univariate Cox regression analysis was adopted to determine the prognostic significance of RBPs from the modules identified by WGCNA, which was conducted in R using “survival” package. And based on LASSO [26, 27] Cox regression algorithm, RBPs with prognostic value were selected to build the risk prediction model using the package “glmnet” in R. The penalty parameter (λ) was determined as per the minimum partial likelihood deviance criteria. The formula below was used to compute the risk score for individual: $\text{risk score=}{\sum }_{\text{j=1}}^{\text{n}}{coef}_{j}\text{*}{x}_{j}$, where Coef_j_ represents the coefficient, whereas X_j_ indicates the relative expression level of each RBPs. Next, we stratified the patients into 2 risk groups (high- and low-risk groups) according to the median risk score. To explore the distribution of different groups, we conducted PCA via the “prcomp” tool of the “stats” R package. The Kaplan-Meier (KM) approach with a log-rank test was employed to evaluate differences between the 2 risk groups in terms of overall survival, which was conducted in R using “survminer” package. Furthermore, the predictive efficacy of this novel model was explored by considering the AUC of the ROC curve through the “survivalROC” package in R. The independent prognostic prediction potential of the risk score was evaluated by Multivariate Cox analysis using “survival” package. Moreover, the relationship of the risk score with the clinicopathological parameters was examined. The mean risk score values of patients in different clinicopathological groups were compared using “stat_compare_means” function in “ggpubr” package. And the method parameter was set as “wilcox.test”. Finally, the prognostic prediction models were validated in an independent GEO CRC cohort (GSE38832). The validation process was to repeat the above experiments in GSE38832.

### Enrichment analyses

In this study, comprehensive enrichment analyses covering 4 aspects were conducted. First, the “clusterProfiler” R package was utilised to perform KEGG along with the GO enrichment analyses targeting the RBPs containing different RBDs (canonical RBDs or non-canonical RBDs). Next, KEGG and GO analyses were also performed regarding distinct modules which were significantly correlated with prognosis identified by the WGCNA. Thirdly, to elucidate the mechanism underlying our prognostic model, GSEA (V.4.1.0, http://software.broadinstitute.org/gsea/) was employed to assess BP, CC, MF and KEGG enrichment based on differently expressed genes between different risk groups predicted by our novel prognostic models (FDR < 0.001, |NES| > 2). Finally, emerging literature have demonstrated the relationship between RBPs and immune status. Therefore, we further used ssGSEA to quantify the enrichment scores of diverse immune cell subpopulations and related functions or pathways. The infiltrating score of 16 immune cells and the activity of 13 immune-related functions or pathways were calculated with ssGSEA in the “gsva” R package. And the NES scores of different risk groups were compared using Wilcoxon method.

### Development of nomogram

As a robust tool to quantify individuals’ risk in a real clinical scenario by integrating multiple risk factors, a nomogram was applied [28–30]. After removing the cases without complete clinical information (447 patients reserved), risk score as well as the clinicopathological parameters (age, gender, stage and TMN) were integrated to establish a nomogram for estimation of one- and three-year OS possibility of individuals with CRC, using the R “survival” and “rms” packages. Moreover, calibration plots were employed to explore the congruency between the estimated and actual survival.

### Identification of candidate drugs

We used the CMap [31] web data resource (https://clue.io) to identify potential candidate drugs. CMap comprises a chemical genomics web data resource that contains gene expression patterns from grown human cells treated with small biomolecules. It can be employed to determine small biomolecules, which revert a distinct gene expression trend. For the potential biomolecules identified by CMap, we examined their drug activity levels from all NCI60 experiments in the CellMiner website (https://discover.nci.nih.gov/cellminer/home.do) and conserved those who passed the quality control assessment.

### External experiments verification of hub prognostic gene expression in final model

The UALCAN online tool (http://ualcan.path.uab.edu/index.html) and the Human Protein Atlas databased (https://www.proteinatlas.org/) were used to validated the expression of 12 genes used in the final model at the transcriptional and translational level. And genetic alterations for these 12 genes were explored with the cBioportal database (http://www.cbioportal.org/).

Fifteen pairs of surgically resected CRC tissue specimens were obtained from Xijing Hospital (Xian, Shanxi Province) and used to detect the mRNA expression levels of the 2 hub genes (TDRD5 and GPC1) identified by our PPI network. Total RNA was extracted from tissue with Trizol reagent (Invitrogen, USA) and cDNA was synthesized by using PrimeScript RT reagent Kit (TaKaRa). The RT-PCR analysis was performed with the SYBR Green PCR Master Mix (TaKaRa) and the ABI StepOne Real-Time PCR system. The mRNA expression levels were normalized to the expression of GAPDH. The primer sequences were TDRD5 forward: 5’-CAACCCTAGACCAGTCCTGT-3’; reverse: 5’-AGTGGACCGATACCCAAGGA-3’; GPC1 forward: 5’- GAGGCTGGTGGCTGCTATG-3’; reverse: 5’- GCAGGTGCTCACCCGAGAT-3’; GAPDH forward: 5’- GACAGTCAGCCGCATCTTCT-3’; reverse: 5’- GCGCCCAATACGACCAAATC-3’. The relative expression of the target gene was calculated by 2^−△△Ct^ method.

### Statistical analysis

All statistical analyses (DEG analysis; univariate, multivariate, and Lasso-penalised Cox regression analyses; KM survival analyses; ROC curve analysis and Wilcoxon test were performed in the R software 4.0.2. *P* < 0.05 signified statistical significance, with all statistical analyses being two sided. Specifically, “limma” package was employed to conduct DEG analysis and “survival” package was employed to conduct univariate and multivariate regression analyses. Lasso-penalised Cox regression analysis was conducted using “glmnet” package. KM curves were plotted using “survminer” package and was compared using a log-rank test. Finally, ROC curve analysis was conducted using “survivalROC” package.

## Results

**Fig. 1:**
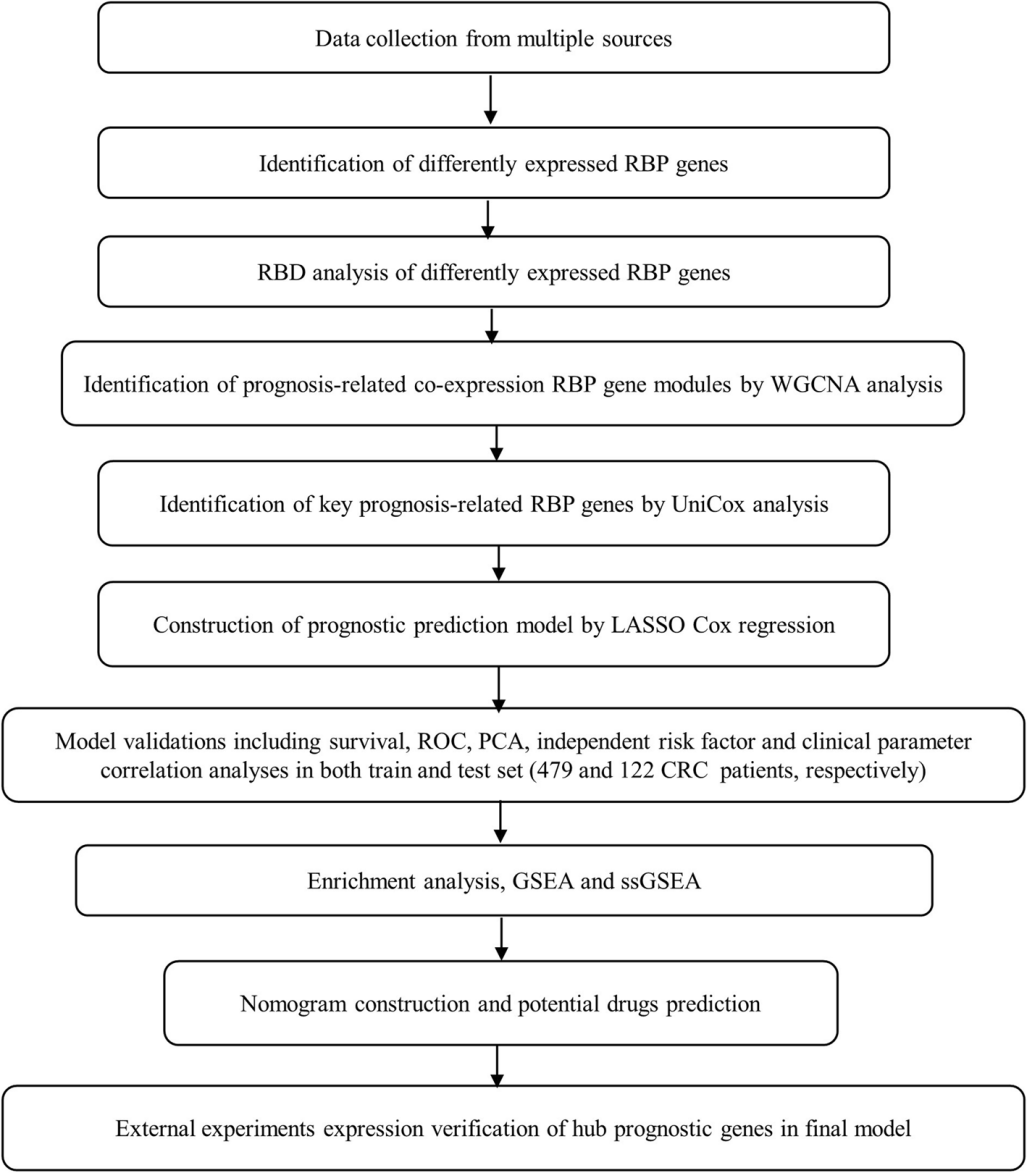
Flow diagram of the study

The flow diagram of the study is displayed in Fig. 1. 479 CRC patients from both TCGA-COAD and TCGA-READ cohorts and 122 CRC patients from the GSE38832 data set were included. The detailed clinical characteristics of these participants are given in Table S2.

### Non-canonical RBPs play an indispensable role in CRC

**Fig. 2:**
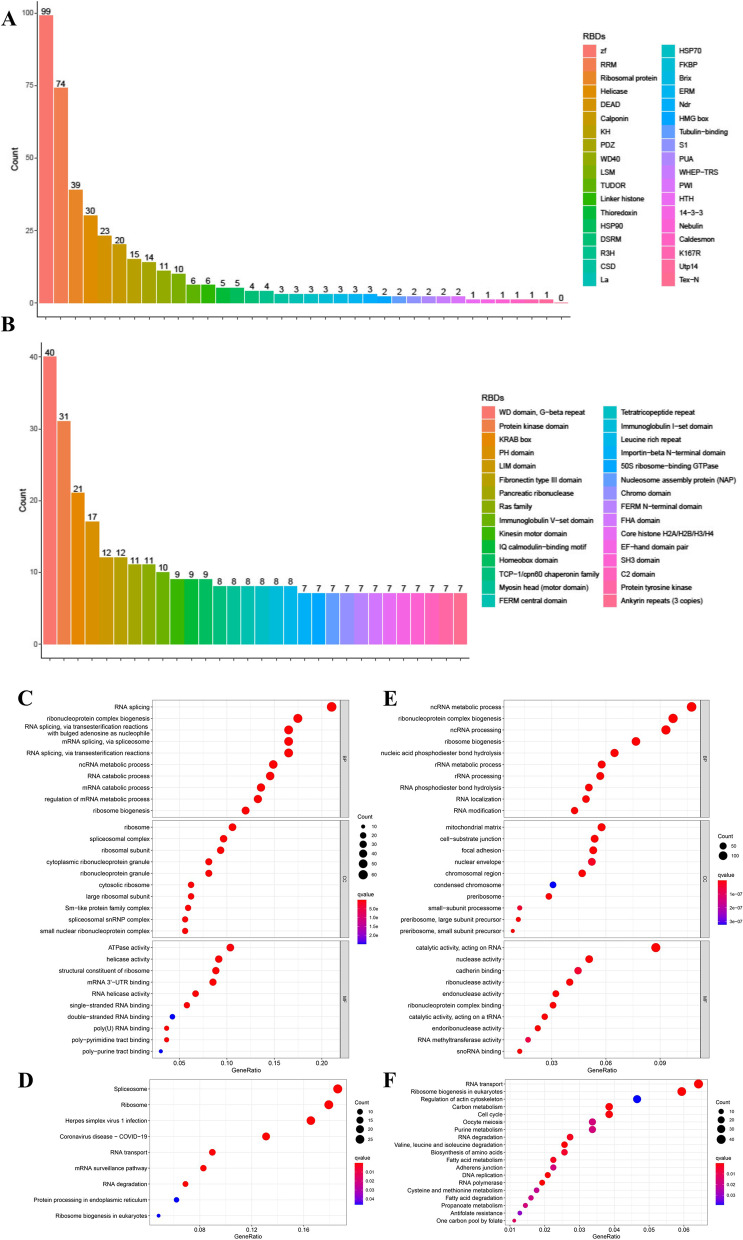
The most enriched RBDs of the 1734 RBPs in the TCGA CRC cohort and enrichment analyses of the RBPs containing different RBDs (canonical RBDs or non-canonical RBDs). **A** Canonical RBDs, (**B**) Non-canonical RBDs. **C** GO along with (**D**) KEGG analyses of canonical RBPs. **E** GO (**F**) along with KEGG analyses of non-canonical RBPs

Different from previous studies, 4082 RBPs (including non-canonical RBPs) were obtained from six resources: Gerstberger, SONAR, the Gene Ontology project, Poly(A)-binding protein, CARIC, and XRNAX. We examined these genes in the TCGA cohort and found that 4001 RBPs had transcriptome data. Among these genes, nearly half of the RBPs (1734/4001, 43.4%) were expressed differentially in cancerous tissue in contrast with the non- cancerous tissue (FDR < 0.05, |logFC|>0.5) (Table S3). Using the David tool, we extracted RBD of the 1734 RBPs. Based on the RBDs, we classified the 1734 RBPs into two categories, including 343 canonical and 1391 non-canonical RBPs. Canonical RBPs contain RBDs which have experimental evidence indicating that they have an RNA-binding function. As shown in Fig. 2A, these canonical RBDs mainly including zinc fingers (ZF), RNA recognition motif (RRM), Ribosomal protein, Helicase conserved C-terminal domain, DEAD/DEAH box helicase (DEAD), Calponin homology (CH) domain, K homology (KH) domain, PDZ domain, tryptophan-aspartic acid 40 (WD40), LSM domain, etc. In non-canonical RBPs, 40 RBPs contain WD domain, G-beta repeat and 31 RBPs contain protein kinase domain (Fig. 2B). But most of the non-canonical RBPs possess distinct functional domains. Therefore, apart from canonical RBPs, non-canonical RBPs may play a different role in CRC. And we utilized “clusterProfiler” R package to perform KEGG along with the GO enrichment analyses regarding these two types of RBPs. Genes contains canonical RBDs were found to be enriched in numerous RNA-related biological processes, for example, RNA splicing and RNA catabolic process. (Fig. 2C). Canonical RBPs also found to be abundant in signalling cascades consisting of spliceosome, ribosome and herpes simplex virus 1 infection (Fig. 2D). As for the RBPs contains non-canonical RBDs, they were also found to be enriched in several RNA-related biological processes such as ncRNA metabolic process, ribonucleoprotein complex biogenesis along with ncRNA processing (Fig. 2E). The enriched signalling pathways were ribosome biogenesis in eukaryotes, RNA transport, DNA replication and RNA polymerase (Fig. 2F). Interestingly, metabolism pathways regarding carbon, fatty acid and amnio acid were also found to be enriched regarding non-canonical RBPs.

### The prognosis-related RBP Gene co-expression modules were identified by WGCNA

**Fig. 3:**
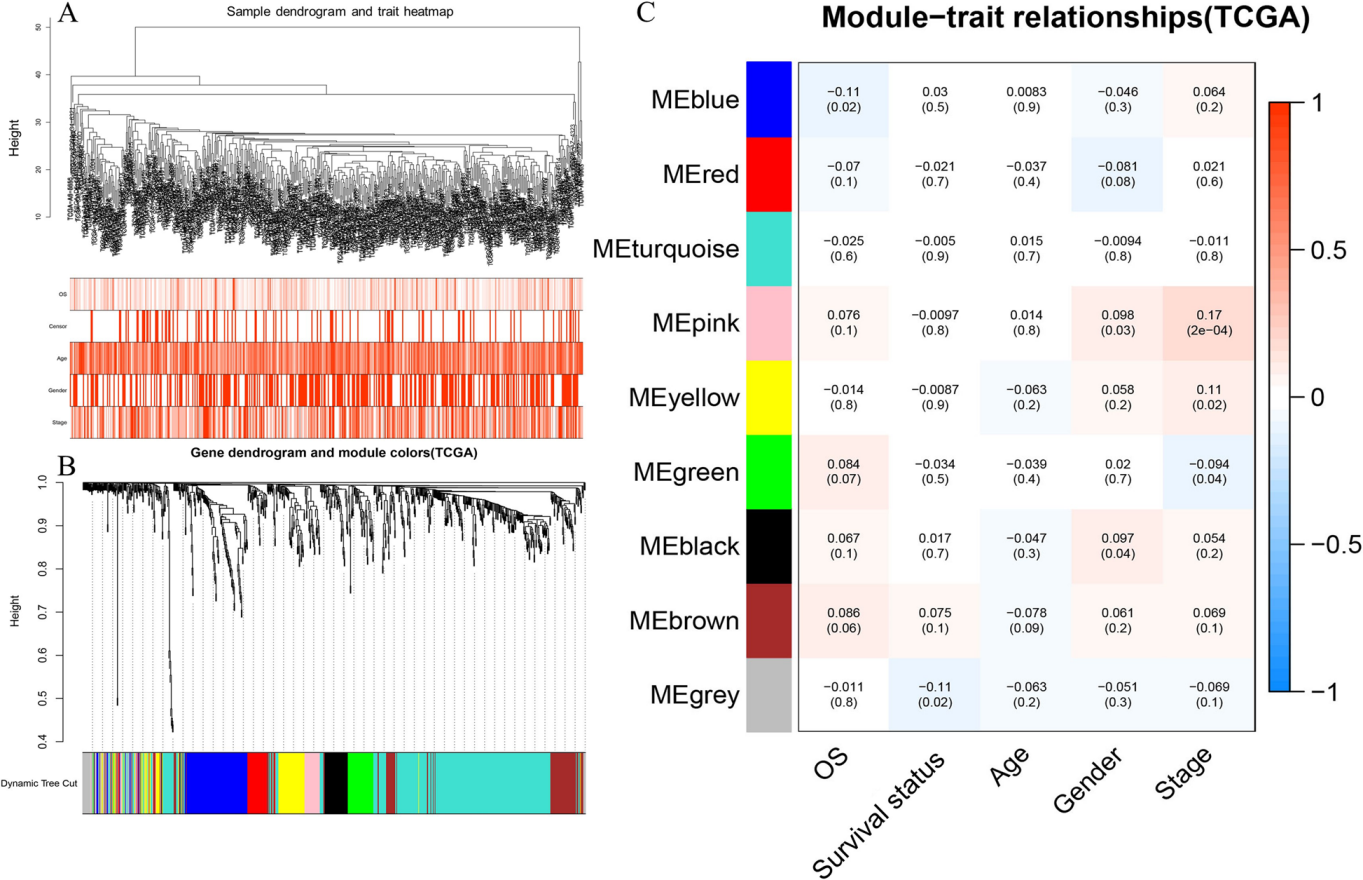
WGCNA analysis of the TCGA CRC cohort. **A** Sample clustering and the correlation with the clinical parameter. **B** Gene clustering and the different co-expressed modules identified by the WGCNA analysis. **C** Correlation between the nine identified co-expression modules and the samples’ clinical trats (OS: overall survival time)

To further determine the most prognosis-associated RBP genes’ co-expression module in CRC, we employed the “WGCNA” package to conduct a gene co-expression network analysis. After clustering, no sample in TCGA was reached the cut-off height 20,000, therefore all 488 tumour samples were utilized in the subsequent analysis (Fig. 3A). A value of β = 5 was employed as the soft-thresholding power to ensure a scale-free network (Figure S1). Overall, 9 modules were uncovered, among which a grey module was automatedly categorised to contain the unassigned genes (Fig. 3B). We also conducted the combination between the similar modules. However, no similarity reaches the threshold and all 9 distinct modules were reserved. Next, the correlation between different modules and clinical traits was analysed (Fig. 3C). Blue module, which contains 113 RBPs, was negatively linked to CRC patients’ OS time (cor = -0.11, *P* < 0.05). Furthermore, another 3 modules, including pink (33 RBPs), yellow (72 RBPs) and green (44 RBPs), were found to be correlated with tumour stage. The pink and yellow module were positively associated with advanced stage, whereas green module was negatively correlated with advanced stage. Because CRC patients with advanced stages usually have a worse prognosis, these 3 modules were also preserved as prognosis-linked modules. Therefore, a total of 4 modules and 262 RBPs were identified for subsequent analysis (Table S4). Scatter plots of the 4 key modules were also shown in Figure S2 to depict the relationship between the gene significance and the gene correlation of their corresponding module.

### Construction and validation of the prognostic signature

**Fig. 4:**
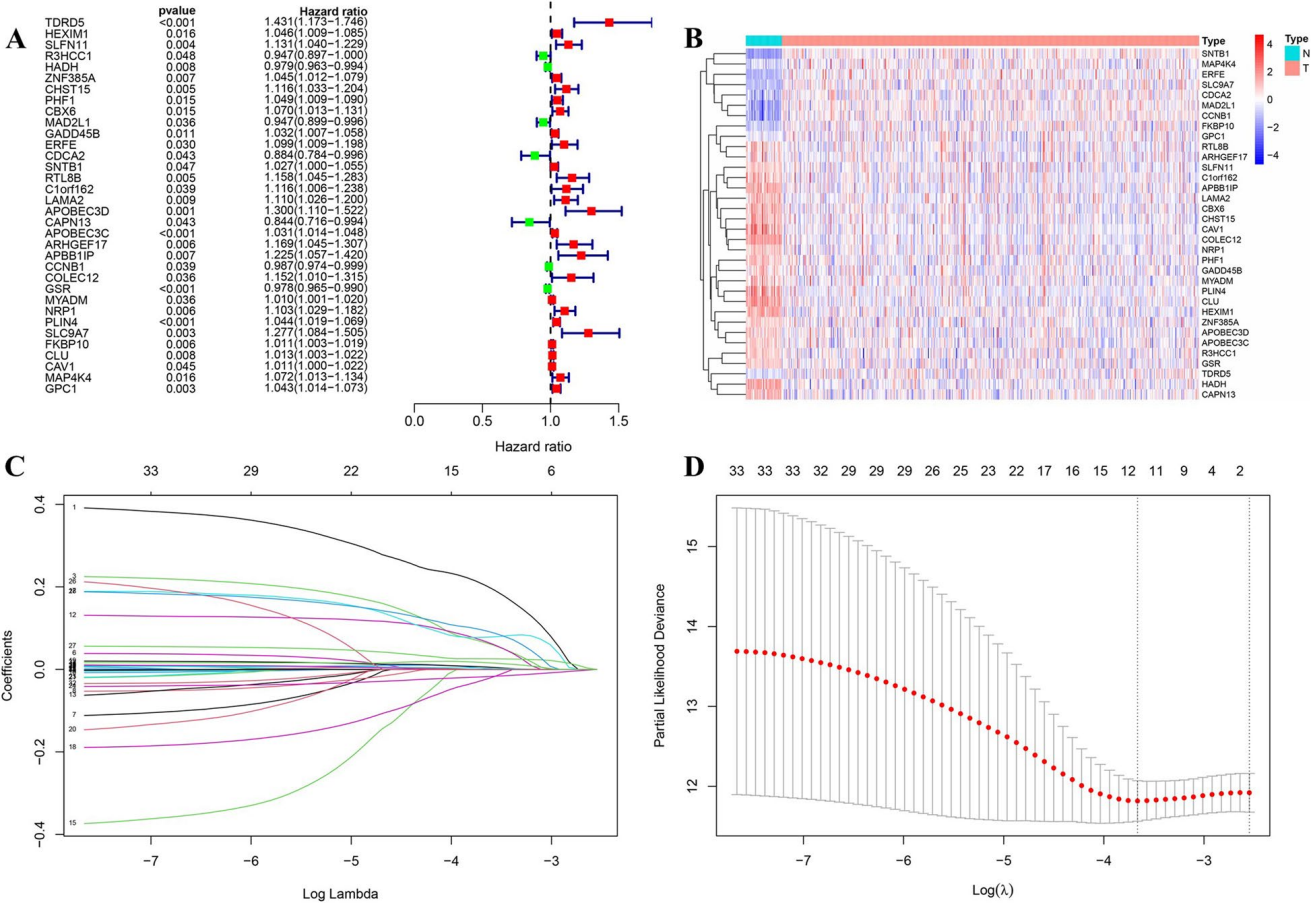
Univariate Cox and Lasso analysis. **A** forest of the 34 identified prognostic RBPs. **B** heatmap of the 34 RBPs between normal and cancer samples. **C** LASSO coefficient profiles of the expression of 34 candidate RBPs. **D** Selection of the penalty parameter (λ) in the LASSO model via cross-validation

Among the 262 genes of the blue, pink, yellow and green modules, we identified 34 significantly prognosis-linked genes via Univariate Cox regression in TCGA cohort (Fig. 4A). The heatmap of these 34 RBPs was shown in Fig. 4B. A predictive gene signature consisting of 12 RBPs was created with the Lasso Cox regression model (Fig. 4C-D). Among the 12 RBPs, 8 came from the blue module, which is correlated with OS time. As for the other 4 RBPs, 2 came from module yellow, 1 came from module pink, and 1 came from module green. These indicated the blue module is the key module for prognosis prediction. The detailed genes in the blue module can be seen in Table S4.

**Fig. 5:**
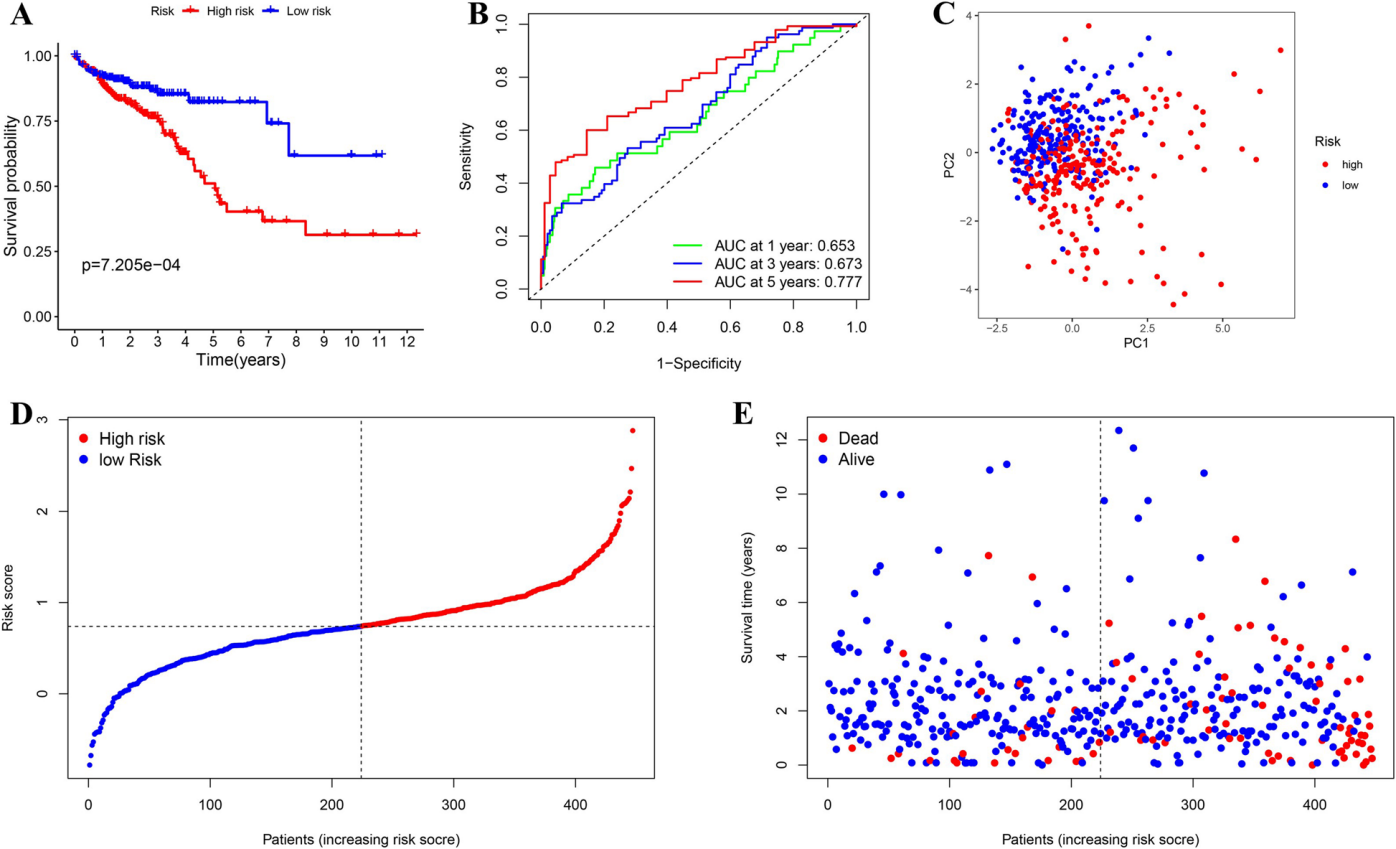
Construction of the prognostic model in the TCGA cohort. **A** Survival analysis of the different risk groups. **B** Time ROC curve of the prognostic model. **C** PCA test of the distribution of the two risk groups. **D** the distribution along with the median value of the risk scores in TCGA data set. **E** the distributions of OS status, OS, as well as the risk score in TCGA data set

Then, the risk score of each patient in the TCGA cohort was calculated based on the formula blow: risk score= (0.204017717772084× expression value of TDRD5) + (0.0651256443619593× expression value of SLFN11) + (0.0680380282919894× expression value of ERFE) + (0.00191741362719572× expression value of LAMA2) + (0.0779271500782849 × expression value of APOBEC3D) + (-0.024608911983806× expression value of CAPN13) + (0.00588789395263925× expression value of APOBEC3C) + (-0.0177344830750462× expression value of GSR) + (0. 0257676876812225× expression value of PLIN4) + (0. 09401945277203× expression value of SLC9A7) + (0. 00298890481343992× expression value of FKBP10) + (0. 016412586214428× expression value of GPC1). A total of 447 patients were categorized into two group on the basis of the median risk score (0.739, Fig. 5D). Kaplan-Meier curves indicated that high-risk patients exhibited worse survival (Fig. 5A), which can also be seen in Fig. 5E. The ROC curves of the predictive signature are shown in Fig. 5B with a 1-year AUC 0.653, 3-year AUC 0.673, and 5-year AUC 0.777. The PCA demonstrated distribution of the patients in the distinct risk groups in two directions (Fig. 5C).

**Fig. 6:**
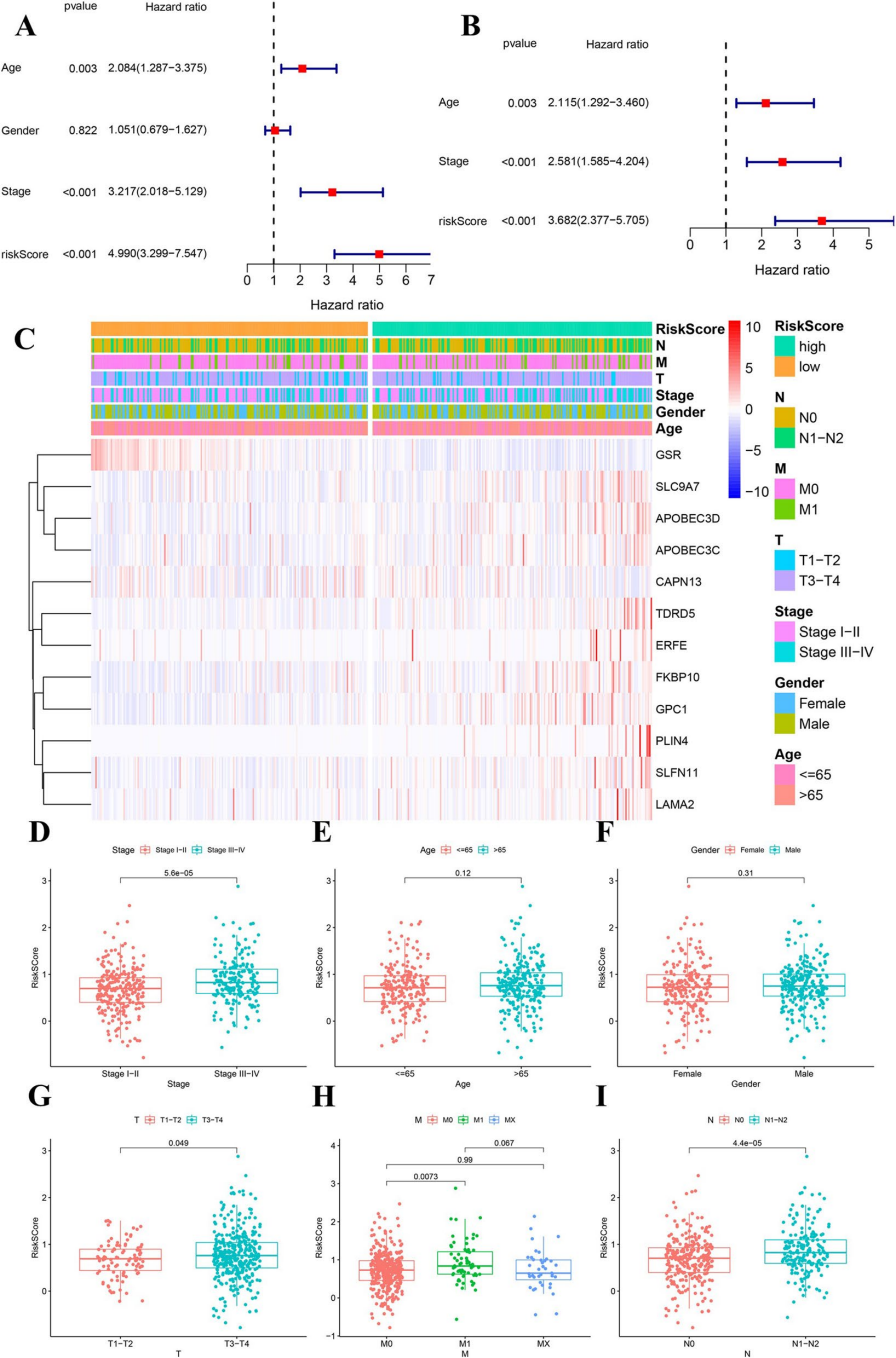
Cox analysis integrating risk score and other clinical parameters and the heatmap of the 12 genes used in our predictive model and correlation between the risk score and clinical parameters in TCGA cohort. **A** Univariate Cox results. **B** Multivariate Cox results. **C** the heatmap of the 12 genes used in our predictive model. **D**-**I** correlation between the risk score and clinical parameters, the clinical parameters from D to I were stage, age, gender, T, M and N, respectively

Univariate Cox analysis suggested that the risk score was significantly associated with OS (Fig. 6A, HR = 4.990, *p* < 0.001), and it was identified as an independent prognostic risk factor by multivariate Cox analysis (Fig. 6B, HR = 3.682, *p* < 0.001). In addition, the risk score was also linked to several clinical parameters such as stage, T, M and N (Fig. 6D-I), which further verified the efficacy of our predictive model.

**Fig. 7:**
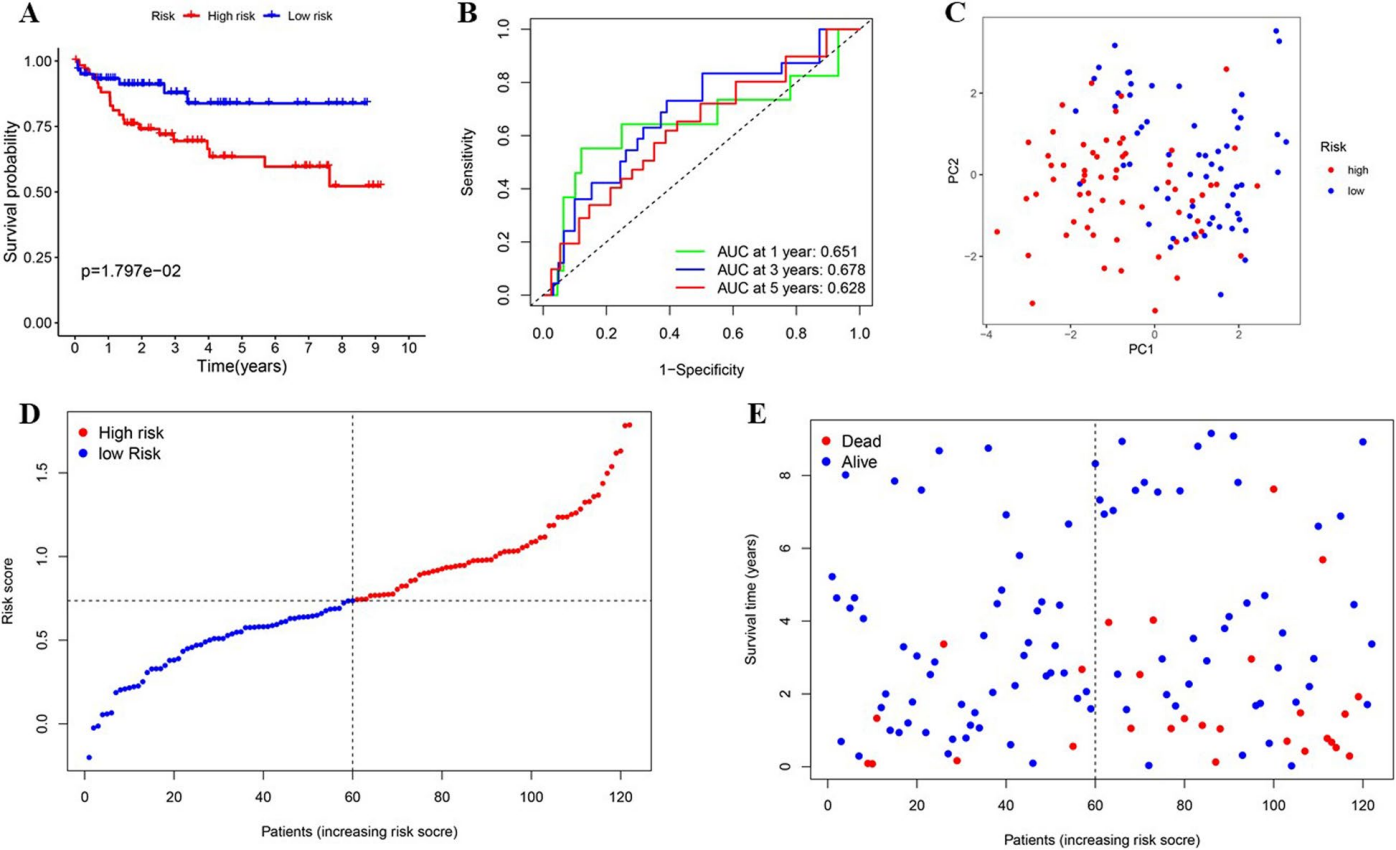
Validation of the prognostic model in the GEO cohort. **A** Survival analysis of the different risk group. **B** Time ROC curve of the prognostic model. **C** PCA test of the distribution of the two risk groups. **D** the distribution along with the median value of the risk scores in GEO data set. **E** the distributions of OS status, OS, as well as the risk score in GEO data set

Next, the model was further verified in an independent CRC dataset GSE38832, which contains 122 CRC patients’ tumour samples. The risk scores of every patient were computed using the same formula above and the 122 patients were classified using the median risk score (Fig. 7D). The results were generally consistent with those found in TCGA cohort. Low risk group were found to have a better chance to live longer (Fig. 7A, E). The ROC curves of the predictive signature were shown in Fig. 7B with a 1-year AUC 0.651, 3-year AUC 0.678, and 5-year AUC 0.628. The PCA demonstrated patients with different risk scores were well distributed in two directions (Fig. 7C). The risk score was also found to be remarkably consistent with tumour stage in GEO cohort (Figure S3).

### A personalised nomogram

**Fig. 8:**
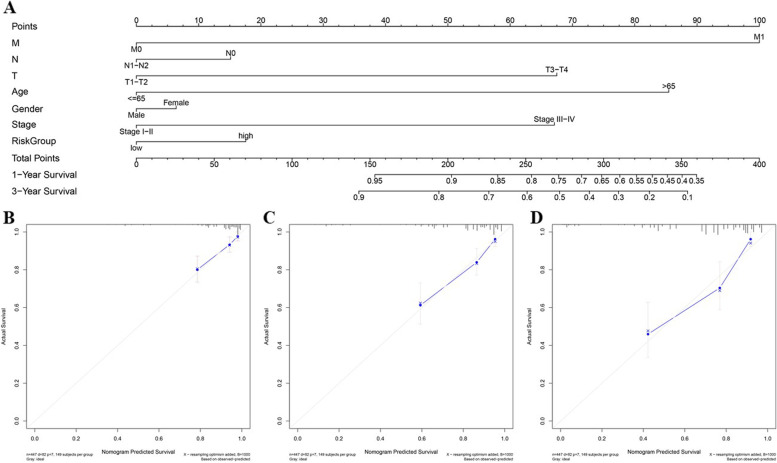
The nomogram to anticipate prognostic probabilities in TCGA-CRC. **A** The nomogram for predicting the OS of TCGA-CRC cohort. **B**-**D** The calibration plots used for predicting one-year (**B**), three-year (**C**), and five-year survival **D**. The x- and y-axes represent predicted nomogram and actual survival, respectively, and the solid line designated the estimated nomogram

A nomogram was constructed to estimate the probability of 1- and 3-year OS by incorporating the 12-RBP gene signatures and other clinicopathological variables, including age, stage, sex and TNM stages. As shown in Fig. 8A, we assigned points to each factor according to its risk contribution to survival. The calibration curves confirmed that actual and estimated survival matched well, especially for 1-year survival (Fig. 8B).

### Enrichment analyses

**Fig. 9:**
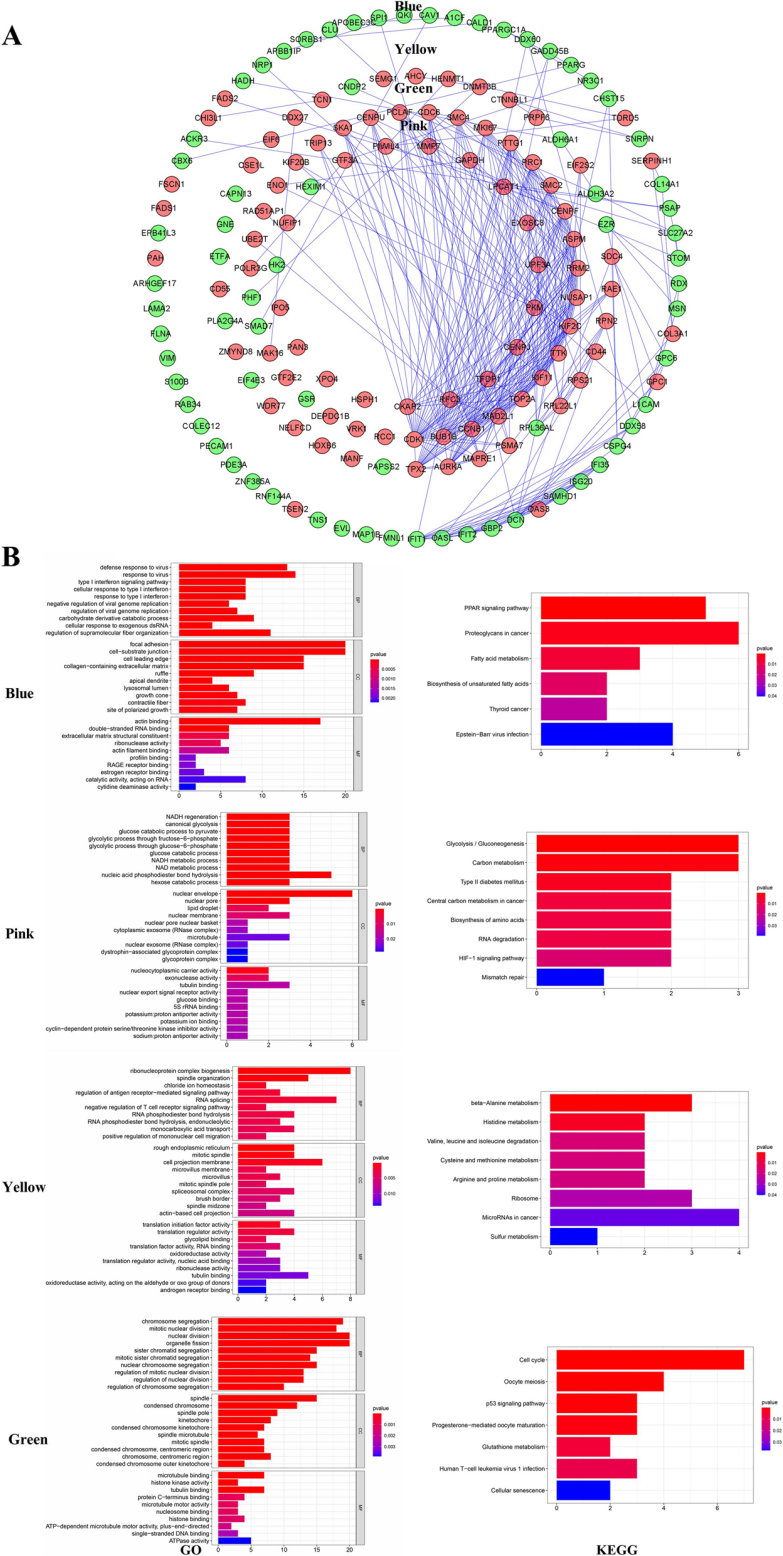
PPI network and enrichment analysis of the 4 prognosis-linked modules identified by WGCNA analysis. **A** PPI network. **B** GO and KEGG enrichment analysis. (Blue circle indicated the genes used in our prognostic model. Red circle exhibits upregulated genes in the cancerous tissue whereas green circle denotes the opposite.)

Firstly, 4 modules correlated with prognosis were identified by WGCNA analysis. The PPI network of these 4 modules was shown in Fig. 9A (cut-off confidence = 0.9), the genes used in our final prognostic module were identified using blue circle. TDRD5 and GPC1 were screened as hub genes, which were upregulated in the cancerous tissue. GO and KEGG analysis for these 4 modules were conducted using the “clusterProfiler” R package (Fig. 9B).

GO analysis revealed that blue module was found to be mainly abundant in response to virus and response to type I interferon (IFN-I) biological processes. The pink module was found to be enriched in several biological processes, such as NADH regeneration and canonical glycolysis. The yellow module was found to be abundant in biological processes consisting of ribonucleoprotein complex biogenesis, RNA splicing and RNA phosphodiester bond hydrolysis. The green module could be categorized into some essential biological processes, including nuclear division, organelle fission and chromosome segregation (Fig. 9B).

KEGG analysis revealed that blue module was mainly associated with PPAR signalling pathway, proteoglycans in cancer and fatty acid metabolism. Pink module was found to be mainly enriched in Glycolysis / Gluconeogenesis, carbon metabolism, RNA degradation and mismatch repair. Yellow module was mainly linked to beta-alanine metabolism and histidine metabolism. Green module was found to be mainly enriched in cell cycle, oocyte meiosis as well as p53 signalling pathway (Fig. 9B).

**Fig. 10:**
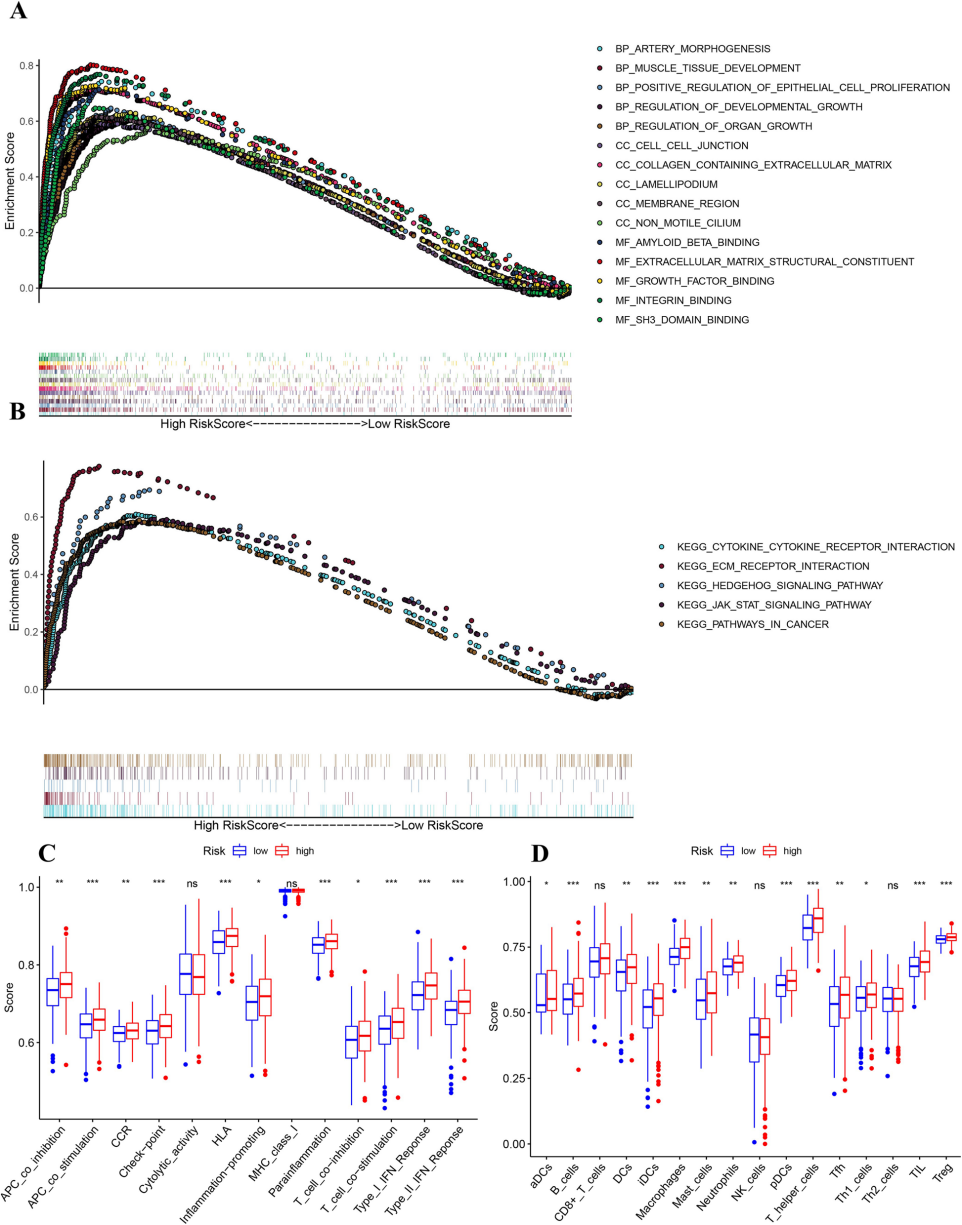
GSEA and ssGSEA analysis of the 12-gene signature between high and low risk groups. **A** GO analysis and (**B**) KEGG analysis based on differently expressed genes between 2 risk groups stratified by the prognostic model. The comparison of the ssGSEA NES scores of (**C**)13 immune-related functions and (D) 16 immune cells between different risk groups in the TCGA cohort were displayed in boxplots. (*P* values were showed as: ns, not significant; *, *P* < 0.05; **, *P* < 0.01; ***, *P* < 0.001.)

Gene set enrichment analysis (GSEA) was performed to obtain a more in-depth insight into biological roles of the prediction signature. Figure 10 A indicated that genes upregulated in the high-risk group were enriched in several essential biological processes such as artery morphogenesis, development of muscle tissue and positive modulation of proliferation of epithelial cells; cellular components such as cell-cell junction and collagen containing extracellular matrix; molecular functions such as amyloid beta binding, extracellular matrix structural component, growth factor binding, integrin binding and SH3 domain binding. Figure 10B shows a few cancer-linked pathways were enriched in high-risk group, including cytokine-cytokine receptor interaction, ECM receptor interaction, HEDGEHOG signalling pathway and JAK/STAT signalling pathway. Finally, ssGSEA analysis revealed the significant differences in the immune scores between the high- and low-risk groups. (Fig. 10C-D). Immune cells including aDCs, B cells, DCs, iDCs, Macrophages, Mast cells, Neutrophils, pDCs, T helper cells, Tfh, Th1 cells, TIL and Treg have a higher infiltration level in the high-risk group. As for the immune-related functions, all of them except for cytolytic activity and MHC class I scored higher on the high-risk group.

### Noscapine and clofazimine were identified as potential drugs by CMap

We first identified 1021 DEGs (135 downregulated, 886 upregulated) between the high- and low-risk groups using “limma” package in R (Table S5, FDR < 0.05, |logFC|>0.5). Using these DEGs as drug targets in CRC, we explored the CMap database to identify small compounds as potential drugs. Table 1 listed the 13 most significant small molecule drugs with potential therapeutic effect on reversing the CRC high-risk gene expression pattern revealed by our signature (cut-off score < -80). The detailed chemical structures of these compounds were indicated in Figure S4. Next, drug activity levels were analysed using the CellMiner web data resource. Among the thirteen candidate drugs, only four (noscapine, orantinib, androstanol and clofazimine) had information in the CellMiner web data resource. The Z scores of the drug activities among the NCI60 cell lines were indicated in Figure S5, and only the drugs with Z scores in the range of 1.2 were mentioned. Noscapine and clofazimine were sensitive in most CRC cells, which were screened as potential drugs for high-risk patients.

**Table 1:** Potential drugs identified by CMap database

Name	Score	Target	MOA (mechanism of action)
isoliquiritigenin	-96.41	AKR1B1, HRH2, SIRT1	Guanylate cyclase activator
beta-CCP	-95.98	GABRA1, GABRG2, IDO1	Indoleamine 2,3-dioxygenase inhibitor
piperacillin	-91.66		Bacterial cell wall synthesis inhibitor
memantine	-87.22	GRIN1, CHRFAM7A, CYP2E1, DRD2, GRIN2A, GRIN2B, GRIN3A, HTR3A	Glutamate receptor antagonist
noscapine	-86.38	BDKRB2, SIGMAR1	Bradykinin receptor antagonist, Tubulin inhibitor
huperzine-a	-86.28	ACHE	Acetylcholinesterase inhibitor
orantinib	-85.89	PDGFRB, AURKA, AURKB, KDR, EGFR, FGFR1, FGFR2, PDGFRA, TBK1	FGFR inhibitor, VEGFR inhibitor, PDGFR receptor inhibitor
androstenol	-85.18	NR1I3	GABA receptor modulator
taurodeoxycholic-acid	-84.95		Bile acid
eicosatetraynoic-acid	-83.34	ALOX12, PPARA, PPARG, PTGS1	Cyclooxygenase inhibitor, Lipoxygenase inhibitor
clofazimine	-82.31		GK0582 inhibitor
norepinephrine	-82	ADRA1A, ADRA1B, ADRA1D, ADRA2A, ADRA2B, ADRA2C, ADRB1, ADRB3, ADRB2, DRD1, DRD5, PAH, SLC18A1, SLC18A2	Adrenergic receptor agonist
vinburnine	-80.09	CHRM1, CHRM2, CHRM3, CHRM4	Adrenergic receptor antagonist

### External validation of the prognostic genes

**Fig. 11:**
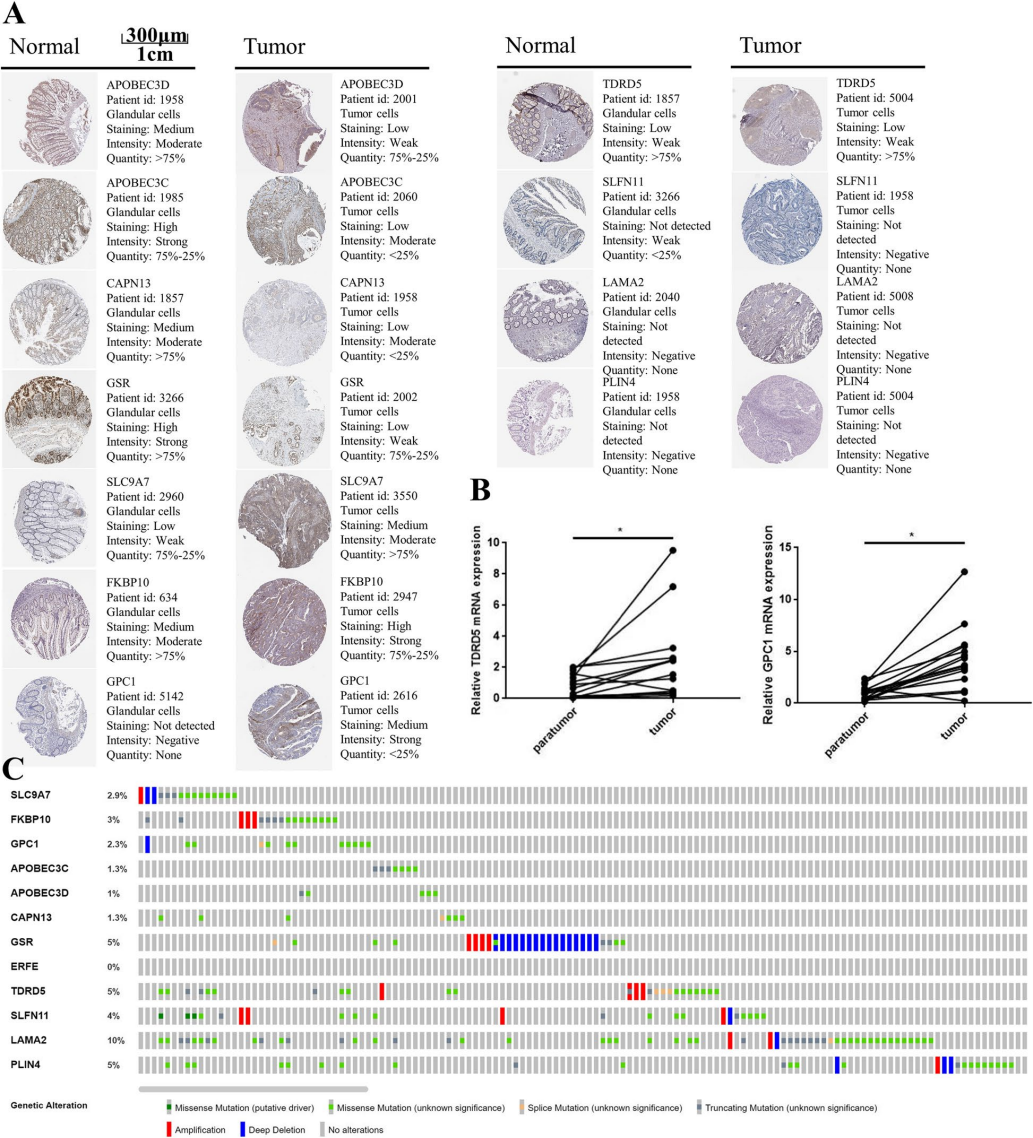
Protein, mRNA expression and genetic alterations of the model genes. **A** Human Protein Atlas database representative protein levels, (**B**) mRNA expression levels of TDRD5 and GPC1 (15 pairs of tissue), (**C**) genomics genetic alterations in CRC using cBioportal

The UALCAN online tool was explored to verified the mRNA expression levels of the 12 genes used in the prediction model. The results were found to be consistent with our DEGs analysis (Figure S6). Then, the protein expression levels were validated using The Human Protein Atlas. Figure 11 A showed that SLC9A7, FKBP10 and GPC1 were overexpressed in CRC tumour tissue compared with normal tissue, whereas APOBEC3C, APOBEC3D, CAPN13 and GSR showed the opposite trend. As for the other 5 genes in the prognostic model, protein expression of ERFE was not found and other 4 genes show no discrepancy in protein expression. In addition, genetic alterations of the 12 prognostic genes were shown in Fig. 11C. LAMA2, GSR, PLIN4 and TDRD5 showed the most frequent alterations. Finally, 15 pairs of CRC samples were collected to validate mRNA expression of the two hub genes (TDRD5 and GPC1) identified by the PPI network in Fig. 9A. Our results showed that TDRD5 and GPC1 were overexpressed in CRC tissue (Fig. 11B). The overexpression of the TDRD5 may attribute to the abnormal genetic amplification (Fig. 11C). In addition, the results of the Cox analysis indicated that the upregulation of TDRD5 and GPC1 was linked with poor overall survival of CRC patients (Fig. 4A.) These results indicated that TDRD5 and GPC1 may serve as potential prognostic biomarkers for CRC patients.

## Discussion

Although diverse genetic drivers and distinct prognostic factors have been broadly explored, patients with CRC remains poor survival. Recent studies have demonstrated that dysregulation of RBPs was significantly correlated to malignant progression in CRC [32]. Hence, this study aims to investigate RBPs’ prognostic value in CRC and propose a novel prediction signature.

Firstly, compared with the other researches that only explored RBPs from traditional sources, our study integrated 4082 RBPs from six resources and investigated their functions based on RBDs. Differently expressed RBPs were categorized into two types based on the RBDs they contained (canonical or non-canonical RBPs). We demonstrated that those two types of RBPs could modulate the progress of CRC via different mechanisms through enrichment analysis. Canonical RBPs primarily participated in RNA splicing, catabolic or metabolic processes, degradation, transportation, and ribosome-related functions. As for non-canonical RBPs, they were associated with several cellular processes, including RNA transport, cell cycle, DNA replication and so on. Non-canonical RBPs were also linked to cell metabolism, such as carbon metabolism, fatty acid metabolism and pyruvate metabolism. In the 12-gene signature we proposed, only 2 genes (TDRD5 and FKBP10) have canonical RBDs, indicating that no-canonical RBPs play an indispensable role in CRC prognosis and larger studies covering all RBP sources rather than canonical ones are needed.

Next, 9 gene co-expression models and the relationships between them and clinical characteristics were determined using WGCNA. Altogether, a total of 4 prognosis-related modules containing 262 RBP genes were identified. As a novel method, WGCNA can identify the key prognostic genes in a co-regulated gene network level instead of an individual gene level, which is more compliant with biology laws. This will make it easier to understand the mechanism underlying the prediction model and find potential therapeutic targets. By measuring the levels of these RBPs, it is perspective to predict the prognosis of individuals with CRC. Therefore, Univariate Cox analysis as well as Lasso algorithm were performed to construct an OS prediction model containing 12 RBP genes (TDRD5, SLFN11, ERFE, LAMA2, APOBEC3D, APOBEC3C, CAPN13, GSR, PLIN4, SLC9A7, FKBP10 and GPC1). The calculated risk score significantly stratified TCGA patient outcomes (*P* < 0.05). Both ROC curves and AUCs validated the efficacy of the prognostic prediction model, which was further verified in an independent GEO dataset (GSE38832). This signature may optimize the individualized survival prediction of CRC patients.

Among these 12 RBP genes used in our model, TDRD5 and GPC1 were uncovered as hub genes by the PPI network as shown in Fig. 9A. Canonical RBP TDRD5 is a member of the Tudor Domain Containing family which encode a group of conserved proteins involved in the spermiogenesis [33]. Mounting evidence has demonstrated that methylated TDRDs can take part in RNA metabolism, alternative splicing, and small RNA pathways [34, 35]. It was reported that hepatocellular carcinoma patients with high expression of TDRD5 suffered poor survival [36]. A previous study conducted by Xuehui Fan et al. also suggested elevated expression of TDRD5 was a risk factor for CRC patients [16]. Another study indicated that mutations and intratumorally heterogeneity of TDRD genes affected the tumorigenesis in microsatellite instability CRC [37]. In our research, TDRD5 was remarkably upregulated in tumour samples and was identified as a prognostic factor for CRC patients (*P* < 0.001, HR = 1.431, CI:1.173–1.746)). As for GPC1, it plays an indispensable role in the control of cell division along with growth modulation. Previous studies revealed that the increased plasma GPC1 + exosomes as well as decreased miR-96-5p and miR-149 were biomarkers for the diagnosis of CRC and a potential therapy target especially for stage III CRC [38, 39]. Literature also identified GPC1 as an independent risk factor in pancreatic ductal adenocarcinoma patients’ prognosis [40]. In our study, GPC1 was remarkably upregulated in tumour samples and was screened as a risk factor for CRC patients’ OS (*P* = 0.003, HR = 1.043, CI:1.014–1.073). The expression of these two hub genes (TDRD and GPC1) were also validated in 15 CRC resected samples. These 2 genes both came from the blue module, which was negatively associated with CRC patients’ OS. Further researches on the role of TDRD5 and GPC1 in CRC progression are needed.

Although it is well established that RBPs were widely involved in regulating CRC biological behaviours, the underlying mechanism remains elusive and needs further investigation. KEGG analysis implied that the prognosis-related blue module (Table S4) identified by WGCNA analysis was remarkedly correlated to PPAR signalling pathway and fatty acid metabolism. Of note, recent study demonstrated that RBP S100A4 promoted M2-like polarization of tumour-associated macrophages via PPAR-γ-dependent fatty acid oxidation, indicating the above pathways may be involved in RBP-mediated CRC progression [41]. Further researches on the blue module are needed. What’s more, our GSEA results suggested that several cancer-linked pathways were enriched in high-risk group predicted by our signature (FDR < 0.001, |NES| > 2), including Hedgehog signalling pathway and JAK/STAT signalling pathway. Previous study reported that enhanced RBP TET1 expression could sensitize pancreatic ductal adenocarcinoma cells to 5FU and gemcitabine through inhibiting the CHL1-related Hedgehog signalling pathway [42]. As for JAK/STAT signaling pathway, recent research demonstrated that RBP CPEB3 could suppress proliferation and migration of CRC cells via binding to 3’UTR of JAK1 mRNA and further inhibiting JAK/STAT pathways [43]. These evidences indicated that 12-RBP gene signature may be implicated in the carcinogenesis of CRC through affecting these signaling pathways, thus contributing to a worse survival in CRC patients. To further explore potential mechanisms of the proposed signature, ssGSEA analysis was conducted. Interestingly, significant correlation was observed between risk score and ssGSEA NES score for tumour infiltrated immune cells (TIICs) and immune function. To our best knowledge, there are inconsistent results in various experiments regarding the correlation of TIICs and the prognosis of CRC individuals [44, 45], which can be attributed to various reasons, for example, different TIICs types, cancer stages, complexed tumour microenvironment (TME) and various cytokines released by tumour cells or TME cells [44]. In our result, high TIICs infiltration level was found in high-risk group (*P* < 0.05). These data suggest that high-risk patients may have higher probability to be benefit from immune therapy and our proposed signature has potential usage in evaluation of immune therapy efficacy in CRC patients. Furthermore, a literature showed a long noncoding RNA VPS9D1-AS1 can amplify intratumoral TGF-β signalling and promote tumour cell escape from CD8 + T cell killing in colorectal cancer by binding a ribosome protein S3 (RPS3) [46], which is a canonical RBP and was overexpressed in CRC tumour samples validated by our DEG analysis (logFC = 0.51, FDR = 4.88E-11). Another paper showed RBP UBE2I may be a diagnostic and surveillance predictive signature for colon cancer and had potential significance of immune infiltrates and promoter methylation [47]. Another research also suggested that RBP YBX3 was associated with tumour immune evasion via different mechanisms involving T-cell exclusion in different cancer types (especially in colon cancer) and by the tumour infiltration of immune cells. And long noncoding RNA HEIH can inhibit this phenomenon by binding with YBX3 [48]. The above literatures suggested some regulatory genes can influence immune cell infiltration and immune cell functions by binding with specific RBPs. Following experiments are needed to determine the relationship between our risk score and immune therapy efficacy.

Lastly, noscapine and clofazimine as potential active drugs for high-risk patients were identified. Noscapine, usually used as a cough suppressant, is a phthalide isoquinoline alkaloid derived from opium. Recently study showed that noscapine could trigger apoptosis in colon cancer cells through the mitochondrial pathways [49]. Another study suggested down-regulation of exogenous CDH17 can enhance apoptosis-triggering impacts of noscapine on CRC [50]. Clofazimine, an anti-mycobacterium drug, could exert antitumor effects through inhibiting Wnt signalling in various cancers, including CRC [51]. A nanoparticulate co-formulation of paclitaxel and clofazimine has been investigated in CRC cells, and was found to be statistically superior to Taxol [52]. Therefore, noscapine and clofazimine may be identified as safe and effective chemotherapeutic agents for the treatment of human CRC, especially for those at high-risk predicted by our model.

## Conclusion

In summary, we profiled the mRNA expression of 4082 RBP genes in TCGA CRC cohort. We conducted WGCNA analysis to screen the most prognosis-related modules and RBP genes and further proposed an OS prediction model based on 12 RBP genes (TDRD5, SLFN11, ERFE, LAMA2, APOBEC3D, APOBEC3C, CAPN13, GSR, PLIN4, SLC9A7, FKBP10 and GPC1), which was determined and validated as an independent prognostic factor for CRC patients. We also constructed a nomogram with good performance in estimating the OS of CRC patients. Finally, two potential drugs were identified. In-depth studies of these hub genes and potential drugs may contribute to personalised therapy for CRC in the clinical setting.

## Electronic supplementary material

**Additional file 1: Table S1. **Literature review regarding bioinformatic analyses of RBP genes in CRC. **Table S2. **Clinical parameters of the TCGA and GEO cohort. **Table S3. **Differently expressed RBP genes between normal and turmor tissues. **Table S4. **The 4 prognosis-related modules identified by WGCNA. **Table S5. **Differently expressed genes between different risk groups. **Figure S1.** Determination of the soft-thresholding powers (β) used in WGCNA. (A) Scale-free fit index and (B) the mean connectivity for various β. **Figure S2.** The correlation between the gene significance for prognostic factor and module membership. Genes in (Aa) blue module, (B) pink module, (C) yellow module and (D) green module. (x represents their correlation with the module, y represents their association with OS or Stage). **Figure S3.** The correlation between the risk score and stage in GEO cohort. **Figure S4.** The chemical structures of the potential drugs identified based on our prognosis model using CMap database (The small compounds’ name from A to M are isoliquiritigenin, beta-CCP, piperacillin, memantine, noscapine, huperzine-a, orantinib, androstanol, taurodeoxycholic-acid, eicosatetraynoic-acid, clofazimine, norepinephrine and vinburnine). **Figure S5.** Drug activities Z-scores among different CRC cell lines calculated by online tool CellMiner. **Figure S6.** mRNA expression levels of the 12 genes validated using UALCAN online tool. (Data of ERFE are not available in this database). 

## Data Availability

All analyzed data related to this paper are included in this paper.

## Abbreviations

RBPs: RNA binding proteins
CRC: colorectal cancer
WGCNA: weighted gene co-expression network analysis
TCGA: The Cancer Genome Atlas
LASSO: Least absolute shrinkage and selection operator
GEO: Gene Expression Omnibus
CMap: Connectivity Map
HPA: Human Protein Atlas
HR: Hazard Ration
CI: Confidence Interval
ROC: Receiver Operating Characteristic Curve
AUC: Area Under Curve
GSEA: Gene Set Enrichment Analysis
ssGSEA: Single Cell Gene Set Enrichment Analysis
RBDs: RNA-binding domains
MEs: module eigengenes
GS: gene significance
OS: Overall Survival
PPI: Protein-protein Interaction Network
KM: Kaplan-Meier
BP: Biological Process
CC: Cellular Component
MF: Molecular Function
UALCAN: The University of Alabama at Birmingham Cancer data analysis Portal
DEG: Differentially Expressed Gene
TME: Tumour Microenvironment.
