# Supplementary material for: Development and validation of an RBP gene signature for prognosis prediction in colorectal cancer based on WGCNA
Source: Hereditas. 2023 Mar 10;160:10. doi: 10.1186/s41065-023-00274-z (PMC9999506; doi:10.1186/s41065-023-00274-z)
Supplement: Supplementary file 1 — Additional file 1: Table S1. Literature review regarding bioinformatic analyses of RBP genes in CRC. Table S2. Clinical parameters of the TCGA and GEO cohort. Table S3. Differently expressed RBP genes between normal and turmor tissues. Table S4. The 4 prognosis-related modules identified by WGCNA. Table S5. Differently expressed genes between different risk groups. Figure S1. Determination of the soft-thresholding powers (β) used in WGCNA. (A) Scale-free fit index and (B) the mean connectivity for various β. Figure S2. The correlation between the gene significance for prognostic factor and module membership. Genes in (Aa) blue module, (B) pink module, (C) yellow module and (D) green module. (x represents their correlation with the module, y represents their association with OS or Stage). Figure S3. The correlation between the risk score and stage in GEO cohort. Figure S4. The chemical structures of the potential drugs identified based on our prognosis model using CMap database (The small compounds’ name from A to M are isoliquiritigenin, beta-CCP, piperacillin, memantine, noscapine, huperzine-a, orantinib, androstanol, taurodeoxycholic-acid, eicosatetraynoic-acid, clofazimine, norepinephrine and vinburnine). Figure S5. Drug activities Z-scores among different CRC cell lines calculated by online tool CellMiner. Figure S6. mRNA expression levels of the 12 genes validated using UALCAN online tool. (Data of ERFE are not available in this database). [file 41065_2023_274_MOESM1_ESM.docx]

**Table S1. Literature review regarding bioinformatic analyses of RBP genes in CRC**

| Aspects | Ours | Zheng Zhang’s | Xuehui Fan’s |
| --- | --- | --- | --- |
| Training Set | 530 samples（42 normal+488 tumor） from TCGA CRC | 612 samples（44 normal+568 tumor） from TCGA CRC | 521(42 tumor+479 normal) from TCGA CRC |
| Independent Validation Set | GSE38832, 122 samples | GSE29623, 130 samples | GSE17536, 177samples |
| External Validations | 1.Correlations between RiskScore and clinical features; | 1.Correlations between expression of four risk genes and clinical features; | None |
|  | 2.HPA database | 2.Timer database |  |
|  | 3. RT-PCR analysis of 15 pairs of CRC samples | 3.HPA database |  |
|  |  | 4.Immunohistochemical staining of 44 pairs of CRC samples |  |
| AUC | Train: (1-year:0.653, 3-year:0.673, 5-year:0.777) | Train: (3-year:0.645, 5-year:0.672) | Train: 0.754 |
|  | Test: (1-year:0.651, 3-year:0.678, 5-year:0.628) | Test: None | Test: 0.553 |
| RBP Source | 4082 from 6 sources | 1542 from 2 sources | 1493, GOA database website |
| RBD analysis | 1394 protein domains of the differently expressed RBPs | None | None |
| OS-related genes identification method | WGCNA+Unicox analysis | Unicox analysis | PPI + Unicox analysis |
| Model Construction Method | LASSO | MultiCox analysis | MultiCox analysis |
| Go and KEGG | 1.Regarding differently expressed RBPs based on different RBDs | Regarding differently expressed RBPs between normal and tumor tissues | 1.Regarding differently expressed RBPs between normal and tumor tissues |
|  | 2.Regarding four modules of prognostic value identify by WGCNA |  | 2.Regarding four key sub-networks identify by the MCODE |
| GSEA | Regarding differently expressed genes between high and low risk groups | None | None |
| ssGSEA | Immune cell infiltration and function were quantified between different risk groups | None | None |
| Potential drug analysis | Noscapine and clofazimine | None | None |
| Genes used in final signature | ***TDRD5***;SLFN11;ERFE;LAMA2;APOBEC3D;CAPN13;APOBEC3C;GSR;PLIN4;SLC9A7;FKBP10;GPC1 | SMAD6;UPF3B;NOL3;PTRH1 | NOP14;MRPS23;MAK16;TDRD6;POP1;***TDRD5***;TDRD7;PPARGC1A;LIN28B;CELF4;LRRFIP2;MSI2 |

**Table S2. Clinical parameters of the TCGA and GEO cohort**

|  | **TCGA Overall** | **GEO Overall** |
| --- | --- | --- |
|  | **(N=476)** | **(N=122)** |
| **Event** |  |  |
| Alive | 384 (80.7%) | 94 (77.0%) |
| Dead | 92 (19.3%) | 28 (23.0%) |
| **Age** |  |  |
| <=65 | 207 (43.5%) |  |
| >65 | 269 (56.5%) |  |
| **Gender** |  |  |
| Female | 220 (46.2%) |  |
| Male | 256 (53.8%) |  |
| **Stage** |  |  |
| Stage I | 85 (17.9%) | 18 (14.8%) |
| Stage II | 180 (37.8%) | 35 (28.7%) |
| Stage III | 126 (26.5%) | 39 (32.0%) |
| Stage IV | 70 (14.7%) | 30 (24.6%) |
| Missing | 15 (3.2%) |  |
| **T** |  |  |
| T1 | 14 (2.9%) |  |
| T2 | 86 (18.1%) |  |
| T3 | 324 (68.1%) |  |
| T4 | 51 (10.7%) |  |
| Missing | 1 (0.2%) |  |
| **M** |  |  |
| M0 | 355 (74.6%) |  |
| M1 | 69 (14.5%) |  |
| MX | 45 (9.5%) |  |
| Missing | 7 (1.5%) |  |
| **N** |  |  |
| N0 | 281 (59.0%) |  |
| N1 | 114 (23.9%) |  |
| N2 | 80 (16.8%) |  |
| Missing | 1 (0.2%) |  |

S

**Table S3. Differently expressed RBP genes between normal and turmor tissues**

|  | **gene** | **conMean** | **treatMean** | **logFC** | **pValue** | **fdr** |
| --- | --- | --- | --- | --- | --- | --- |
| **RBPs** | PDCD4 | 86.79496 | 20.45847 | -2.08491 | 2.91E-26 | 6.77E-24 |
|  | ADD1 | 27.4805 | 13.63281 | -1.01133 | 2.48E-26 | 6.77E-24 |
|  | DDX31 | 1.948195 | 4.305598 | 1.144075 | 2.52E-26 | 6.77E-24 |
|  | RBM28 | 1.140384 | 2.799206 | 1.295498 | 2.79E-26 | 6.77E-24 |
|  | NKRF | 1.807435 | 5.009261 | 1.470654 | 1.64E-26 | 6.77E-24 |
|  | DKC1 | 15.5549 | 40.6684 | 1.386539 | 1.62E-26 | 6.77E-24 |
|  | PUS7 | 2.619654 | 8.47922 | 1.694555 | 2.44E-26 | 6.77E-24 |
|  | NAP1L2 | 1.890218 | 0.130097 | -3.86089 | 1.55E-26 | 6.77E-24 |
|  | EPOP | 0.253672 | 4.899588 | 4.271623 | 8.11E-27 | 6.77E-24 |
|  | B3GNTL1 | 0.316092 | 1.302476 | 2.042841 | 2.45E-26 | 6.77E-24 |
|  | AJUBA | 0.45402 | 4.671925 | 3.363189 | 6.00E-27 | 6.77E-24 |
|  | CHGA | 110.1681 | 5.389564 | -4.35339 | 2.87E-26 | 6.77E-24 |
|  | TRIP13 | 1.637299 | 8.573772 | 2.388612 | 1.67E-26 | 6.77E-24 |
|  | MAMDC2 | 9.187261 | 0.306693 | -4.90477 | 1.40E-26 | 6.77E-24 |
|  | MTHFD1L | 2.052722 | 11.17753 | 2.444991 | 7.97E-27 | 6.77E-24 |
|  | SLC39A10 | 1.621336 | 6.477205 | 1.998188 | 8.39E-27 | 6.77E-24 |
|  | ZC3HAV1L | 1.022546 | 5.302564 | 2.374524 | 3.42E-26 | 7.08E-24 |
|  | EPB41L3 | 10.42105 | 1.39587 | -2.90026 | 3.25E-26 | 7.08E-24 |
|  | HADHB | 54.91687 | 25.45636 | -1.10922 | 4.70E-26 | 8.40E-24 |
|  | NDRG2 | 29.35701 | 9.675758 | -1.60126 | 4.40E-26 | 8.40E-24 |
|  | PACSIN2 | 49.25214 | 20.68686 | -1.25147 | 4.73E-26 | 8.40E-24 |
|  | MMP7 | 0.204944 | 31.70562 | 7.273368 | 7.03E-26 | 1.19E-23 |
|  | AHCYL2 | 86.52497 | 12.08651 | -2.83972 | 1.02E-25 | 1.23E-23 |
|  | CBX8 | 1.215076 | 4.32365 | 1.831203 | 1.09E-25 | 1.23E-23 |
|  | XPO5 | 4.542006 | 10.27081 | 1.177149 | 7.95E-26 | 1.23E-23 |
|  | TOMM34 | 16.31517 | 53.5785 | 1.71544 | 8.45E-26 | 1.23E-23 |
|  | ACAT1 | 27.94765 | 10.16561 | -1.45903 | 1.02E-25 | 1.23E-23 |
|  | MRPL35 | 25.66968 | 13.36735 | -0.94135 | 1.04E-25 | 1.23E-23 |
|  | EIF4E3 | 7.96993 | 1.936618 | -2.04103 | 8.74E-26 | 1.23E-23 |
|  | RAP1A | 28.09512 | 10.55397 | -1.41253 | 1.06E-25 | 1.23E-23 |

*Only show top30 sorted by FDR

**Table S4. The 4 prognosis-related modules identified by WGCNA**

| **Blue** | **Pink** | **Yellow** | **Green** |
| --- | --- | --- | --- |
| TDRD5 | AEN | CD44 | MAK16 |
| QKI | HEXIM1 | TPX2 | RCC1 |
| RDX | RBM26 | MAPRE1 | UBE2T |
| CSRP1 | XPO4 | EZR | PCLAF |
| PARP12 | IPO5 | RPS21 | ENO1 |
| FSCN1 | NUFIP1 | MANF | MKI67 |
| SLFN11 | ARGLU1 | ALDH6A1 | R3HCC1 |
| CALD1 | GAPDH | LSM14B | TOP2A |
| MSN | PKM | MEX3A | NUSAP1 |
| BASP1 | MMP7 | LGALS3 | CENPU |
| ZCCHC24 | UPF3A | WDR77 | PHF1 |
| IFIT2 | CENPJ | STAU1 | MAD2L1 |
| OAS3 | EEF1AKMT1 | RBM39 | ACSF2 |
| VIM | TARBP1 | EIF6 | POLR3G |
| RBPMS2 | PAN3 | DDX27 | PSRC1 |
| OASL | SNTB1 | RPL22L1 | RAD51AP1 |
| TAGLN | GTF3A | PRPF6 | CDCA2 |
| SERPINH1 | GPALPP1 | CSE1L | GTF2E2 |
| HADH | PROSER1 | RPL36AL | SMAD7 |
| MAP1B | PCID2 | MTG2 | TRIP13 |
| DDX60L | SEMA4B | KHDRBS3 | WIPI1 |
| KCTD12 | EXOSC8 | CIRBP | BUB1B |
| ZNF385A | PIWIL4 | EIF2S2 | VRK1 |
| FLNA | RNF219 | FARP1 | CCNB1 |
| S100A9 | MTRF1 | SIDT1 | CDK1 |
| CNN1 | LPCAT1 | PAPSS2 | CDC6 |
| A1CF | RFC3 | TOMM34 | PTTG1 |
| PLEKHO1 | SLC9A7 | DNMT3B | GSR |
| CHST15 | TFDP1 | CNDP2 | PRC1 |
| SAMSN1 | CKAP2 | NELFCD | FAM111B |
| CHI3L1 | HK2 | ERFE | WLS |
| CBX6 | HSPH1 | NECTIN4 | DEPDC1B |
| PPARGC1A | RETSAT | GNE | KIF11 |
| MAP1A |  | GAS2 | SMC4 |
| S100B |  | ST6GALNAC1 | KIF20B |
| EPB41L3 |  | RAE1 | KIF2C |
| CDR2L |  | CTNNBL1 | SKA1 |
| THEMIS2 |  | HENMT1 | SMC2 |
| GADD45B |  | PLEKHB1 | ASPM |
| FMNL1 |  | ERN2 | ATAD2 |
| OGN |  | ANG | RRM2 |
| SAMHD1 |  | CAPN13 | WBP2 |
| SPI1 |  | DMKN | TTK |
| RNASE1 |  | TCN1 | CENPF |
| CTSO |  | YBEY |  |
| DCN |  | HOXB6 |  |
| LDOC1 |  | EIF4E3 |  |
| RTL8B |  | FOXP1 |  |
| EVL |  | GDA |  |
| C1orf162 |  | ALDH3A2 |  |
| LAMA2 |  | AURKA |  |
| APOBEC3D | | ETFA |  |
| PLEK |  | ABCC2 |  |
| RNASE6 |  | TM9SF4 |  |
| RERG |  | CA12 |  |
| CRYAB |  | HM13 |  |
| PPARG |  | ADNP |  |
| RNF144A |  | PIGU |  |
| FADS2 |  | RPN2 |  |
| NR3C1 |  | SEMG1 |  |
| DDX60 |  | SERPINB5 |  |
| GBP2 |  | PROM2 |  |
| AXDND1 |  | CD55 |  |
| TNS1 |  | ZMYND8 |  |
| PNRC1 |  | AHCY |  |
| COL3A1 |  | PLA2G4A |  |
| PECAM1 |  | SDC4 |  |
| SORBS1 |  | S100A14 |  |
| TSEN2 |  | PTK7 |  |
| IFIT1 |  | SLC7A2 |  |
| CORO1A |  | SLC5A6 |  |
| COL14A1 |  | PSMA7 |  |
| SNRPN |  |  |  |
| ISG20 |  |  |  |
| APOBEC3C | |  |  |
| IFI35 |  |  |  |
| MS4A7 |  |  |  |
| ARHGEF17 | |  |  |
| DDX58 |  |  |  |
| CASQ2 |  |  |  |
| GPC6 |  |  |  |
| APBB1IP |  |  |  |
| TNFAIP8L2 | |  |  |
| CLMP |  |  |  |
| CSPG4 |  |  |  |
| SUSD2 |  |  |  |
| FADS1 |  |  |  |
| PALLD |  |  |  |
| FHL1 |  |  |  |
| PDE3A |  |  |  |
| COLEC12 |  |  |  |
| MXRA7 |  |  |  |
| AKAP12 |  |  |  |
| ATP2B4 |  |  |  |
| RAB34 |  |  |  |
| SLC27A2 |  |  |  |
| ACKR3 |  |  |  |
| MYADM |  |  |  |
| NRP1 |  |  |  |
| PLIN4 |  |  |  |
| ANXA6 |  |  |  |
| STOM |  |  |  |
| FKBP10 |  |  |  |
| MCAM |  |  |  |
| TXNIP |  |  |  |
| L1CAM |  |  |  |
| PSAP |  |  |  |
| PLXDC2 |  |  |  |
| CLU |  |  |  |
| CAV1 |  |  |  |
| MAP4K4 |  |  |  |
| GPC1 |  |  |  |
| PAH |  |  |  |

**Table S5. Differently expressed genes between different risk groups**

| **gene** | **lowMean** | **highMean** | **logFC** | **pValue** | **fdr** |
| --- | --- | --- | --- | --- | --- |
| GSR | 48.49493 | 29.00326 | -0.74162 | 8.27E-26 | 1.02E-21 |
| ADPRH | 1.250368 | 2.004025 | 0.680547 | 2.88E-24 | 9.14E-21 |
| SERPING1 | 18.11299 | 38.69748 | 1.095215 | 2.25E-24 | 9.14E-21 |
| ARHGEF25 | 1.1046 | 2.368465 | 1.100429 | 2.96E-24 | 9.14E-21 |
| CLIP3 | 1.848228 | 3.598849 | 0.961393 | 1.53E-23 | 3.67E-20 |
| GLIS2 | 2.031318 | 4.013188 | 0.982333 | 1.78E-23 | 3.67E-20 |
| TIMP2 | 23.00989 | 45.14687 | 0.972372 | 1.01E-21 | 1.58E-18 |
| COL8A2 | 1.267355 | 2.840912 | 1.164534 | 1.02E-21 | 1.58E-18 |
| RTL8B | 1.465549 | 2.826891 | 0.947775 | 1.91E-21 | 2.63E-18 |
| FXYD6 | 1.081417 | 2.542289 | 1.233205 | 2.62E-21 | 3.24E-18 |
| C1R | 17.60516 | 33.17516 | 0.914105 | 3.78E-21 | 4.24E-18 |
| TNS1 | 3.163794 | 7.917544 | 1.323397 | 5.17E-21 | 5.32E-18 |
| CCDC8 | 0.637632 | 1.371198 | 1.104642 | 1.13E-20 | 1.08E-17 |
| RAB3IL1 | 1.987822 | 3.571424 | 0.845311 | 1.59E-20 | 1.23E-17 |
| FBXL7 | 0.736089 | 1.344002 | 0.868583 | 1.55E-20 | 1.23E-17 |
| MRC2 | 5.624582 | 10.93183 | 0.958717 | 1.47E-20 | 1.23E-17 |
| ARHGEF17 | 1.948881 | 3.324031 | 0.770288 | 1.95E-20 | 1.42E-17 |
| MAP1A | 0.787762 | 1.573649 | 0.998283 | 2.38E-20 | 1.63E-17 |
| TNFSF12 | 4.608097 | 7.379664 | 0.679384 | 2.74E-20 | 1.79E-17 |
| GPC1 | 5.148588 | 9.403797 | 0.869067 | 2.98E-20 | 1.84E-17 |
| SSC5D | 1.01424 | 2.532988 | 1.320441 | 3.17E-20 | 1.86E-17 |
| KANK2 | 5.689195 | 9.049002 | 0.669534 | 6.42E-20 | 3.61E-17 |
| MRAS | 0.932131 | 1.740959 | 0.901278 | 6.83E-20 | 3.67E-17 |
| ZNF532 | 1.243415 | 2.177762 | 0.808539 | 7.16E-20 | 3.69E-17 |
| ARMCX2 | 1.502189 | 3.005728 | 1.000648 | 7.76E-20 | 3.84E-17 |
| JAM3 | 1.835344 | 3.044669 | 0.730235 | 8.65E-20 | 3.96E-17 |
| AEBP1 | 29.03654 | 62.02117 | 1.094891 | 8.42E-20 | 3.96E-17 |
| ITGA11 | 1.969775 | 3.881202 | 0.978473 | 9.00E-20 | 3.97E-17 |
| RFTN1 | 3.981154 | 6.595812 | 0.728364 | 1.15E-19 | 4.76E-17 |
| FBLN5 | 2.041102 | 3.886939 | 0.929286 | 1.14E-19 | 4.76E-17 |
| BGN | 73.74308 | 147.2634 | 0.997819 | 1.49E-19 | 5.76E-17 |
| SHISA4 | 1.940676 | 3.68312 | 0.924369 | 1.72E-19 | 6.43E-17 |
| THBS2 | 12.95578 | 29.79623 | 1.201534 | 2.20E-19 | 7.99E-17 |
| GUCY1A1 | 1.41595 | 2.960959 | 1.064294 | 2.76E-19 | 9.46E-17 |
| CCDC80 | 1.941566 | 4.753081 | 1.291643 | 2.70E-19 | 9.46E-17 |
| AOC3 | 2.859139 | 7.080117 | 1.308192 | 3.19E-19 | 1.07E-16 |
| DEPP1 | 6.849728 | 13.93922 | 1.025031 | 4.10E-19 | 1.33E-16 |
| CNTNAP1 | 0.9449 | 1.80778 | 0.935985 | 4.47E-19 | 1.42E-16 |
| CCDC102A | 1.701965 | 2.574591 | 0.597141 | 5.00E-19 | 1.55E-16 |
| C1S | 19.14649 | 36.41177 | 0.927325 | 5.35E-19 | 1.57E-16 |
| ISLR | 16.17658 | 33.16514 | 1.035761 | 5.28E-19 | 1.57E-16 |
| APBB1 | 1.113258 | 2.102885 | 0.917583 | 5.60E-19 | 1.61E-16 |
| PRELP | 1.46419 | 4.325846 | 1.562879 | 6.52E-19 | 1.83E-16 |
| CTSF | 3.880255 | 7.543698 | 0.95912 | 8.47E-19 | 2.33E-16 |
| VIM | 40.76098 | 68.85873 | 0.756451 | 9.35E-19 | 2.46E-16 |
| MYL9 | 40.29016 | 88.58374 | 1.136614 | 9.17E-19 | 2.46E-16 |
| C3 | 13.51753 | 36.26375 | 1.423697 | 1.12E-18 | 2.89E-16 |
| CPQ | 4.046043 | 6.455788 | 0.674081 | 1.38E-18 | 3.40E-16 |
| NXN | 1.901776 | 3.575801 | 0.910919 | 1.37E-18 | 3.40E-16 |
| MSN | 19.79089 | 31.93135 | 0.690137 | 1.62E-18 | 3.78E-16 |

**Only show top30 sorted by FDR


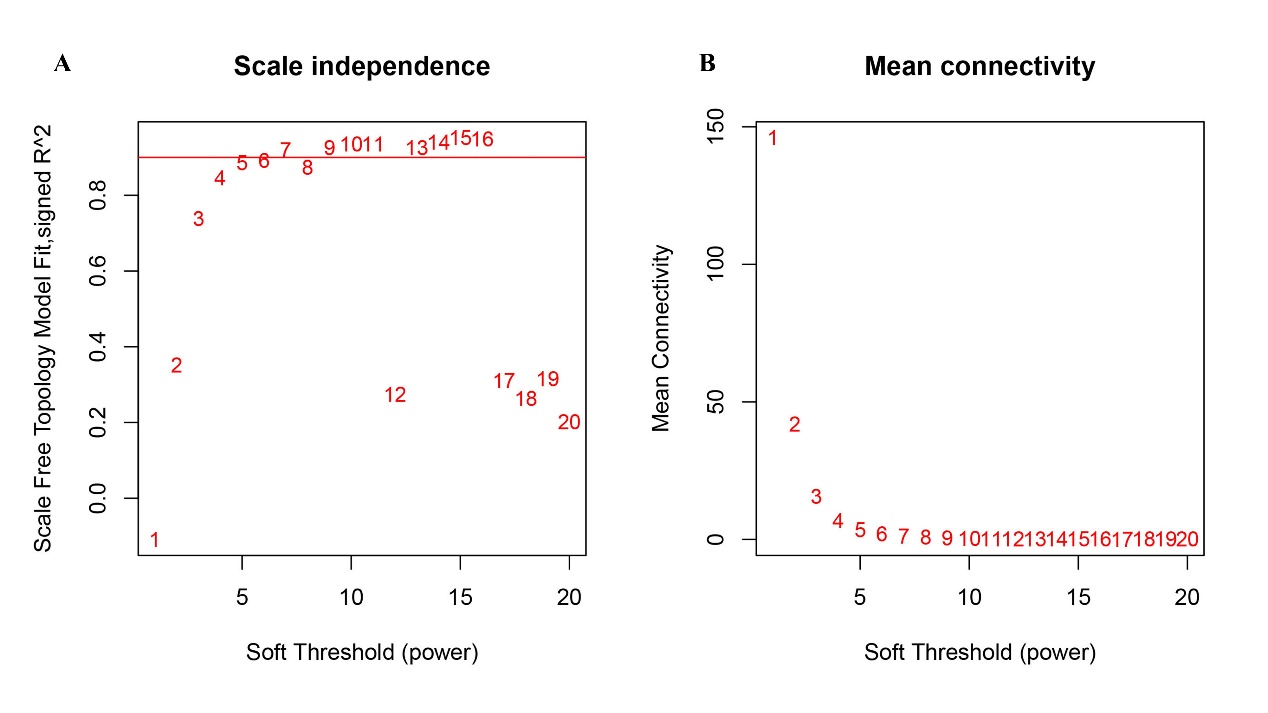


Figure S1. Determination of the soft-thresholding powers (β) used in WGCNA. (A) Scale-free fit index and (B) the mean connectivity for various β.


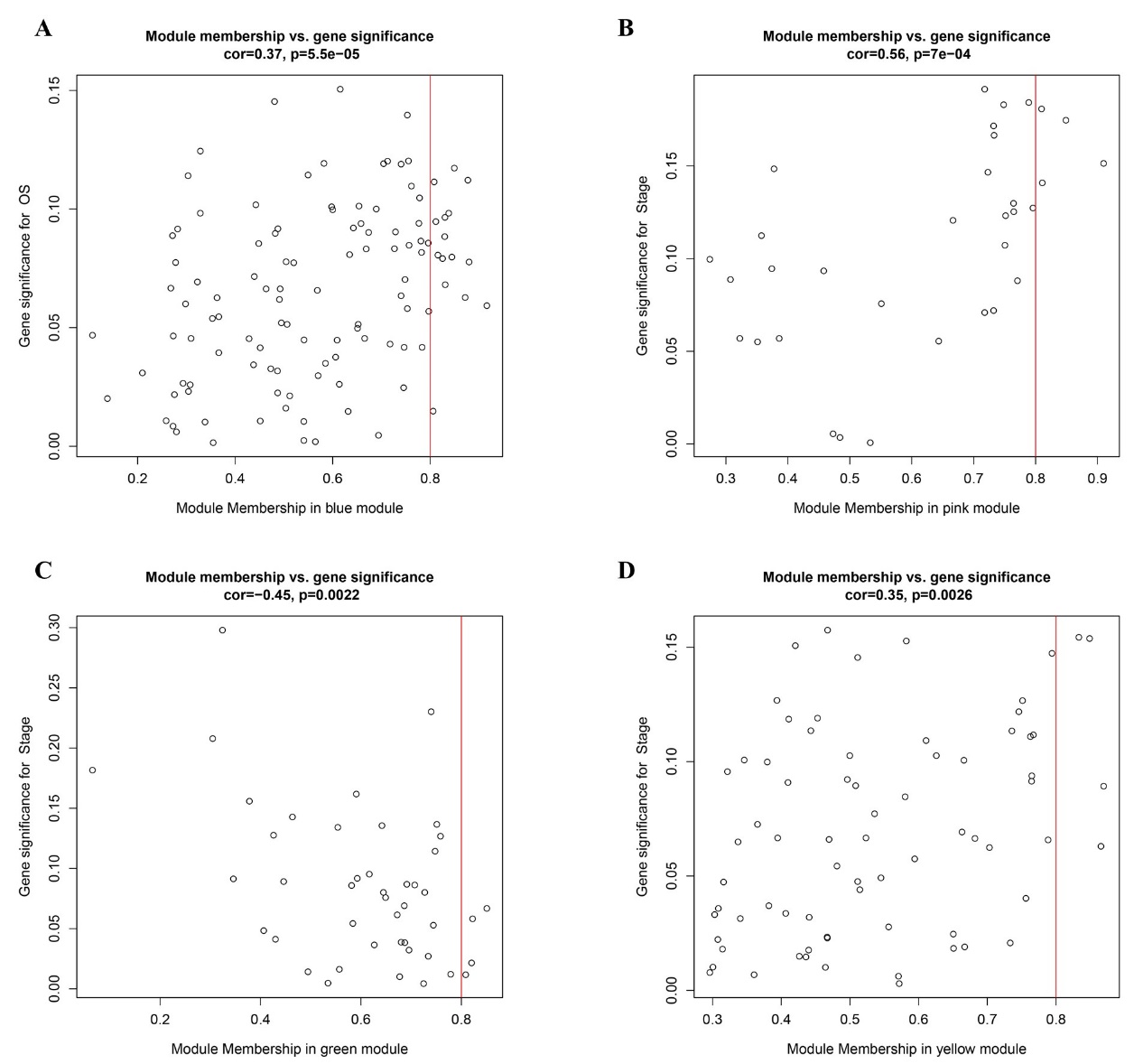


Figure S2. The correlation between the gene significance for prognostic factor and module membership. Genes in (Aa) blue module, (B) pink module, (C) yellow module and (D) green module. (x represents their correlation with the module, y represents their association with OS or Stage).


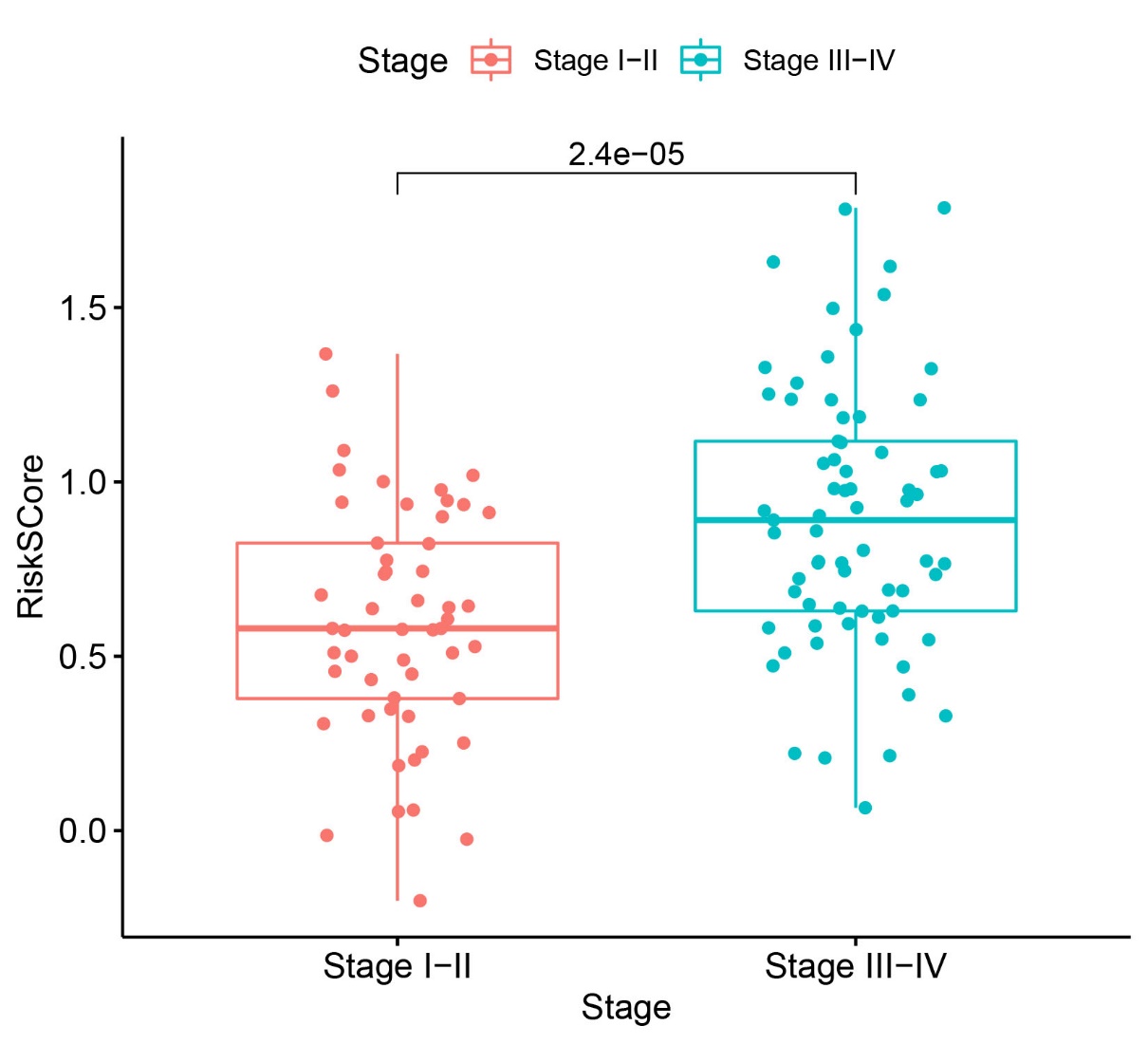


Figure S3. The correlation between the risk score and stage in GEO cohort.


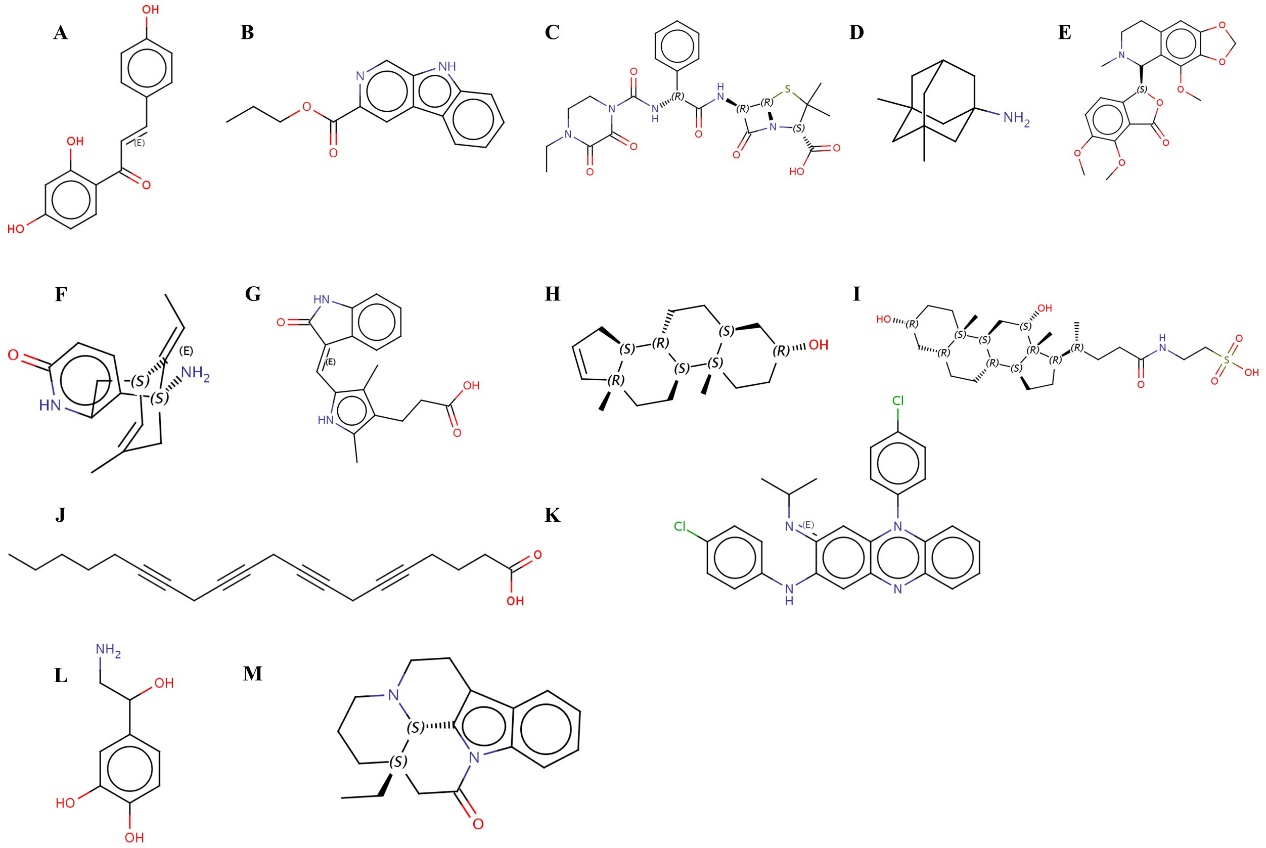


Figure S4. The chemical structures of the potential drugs identified based on our prognosis model using CMap database (The small compounds’ name from A to M are isoliquiritigenin, beta-CCP, piperacillin, memantine, noscapine, huperzine-a, orantinib, androstanol, taurodeoxycholic-acid, eicosatetraynoic-acid, clofazimine, norepinephrine and vinburnine).


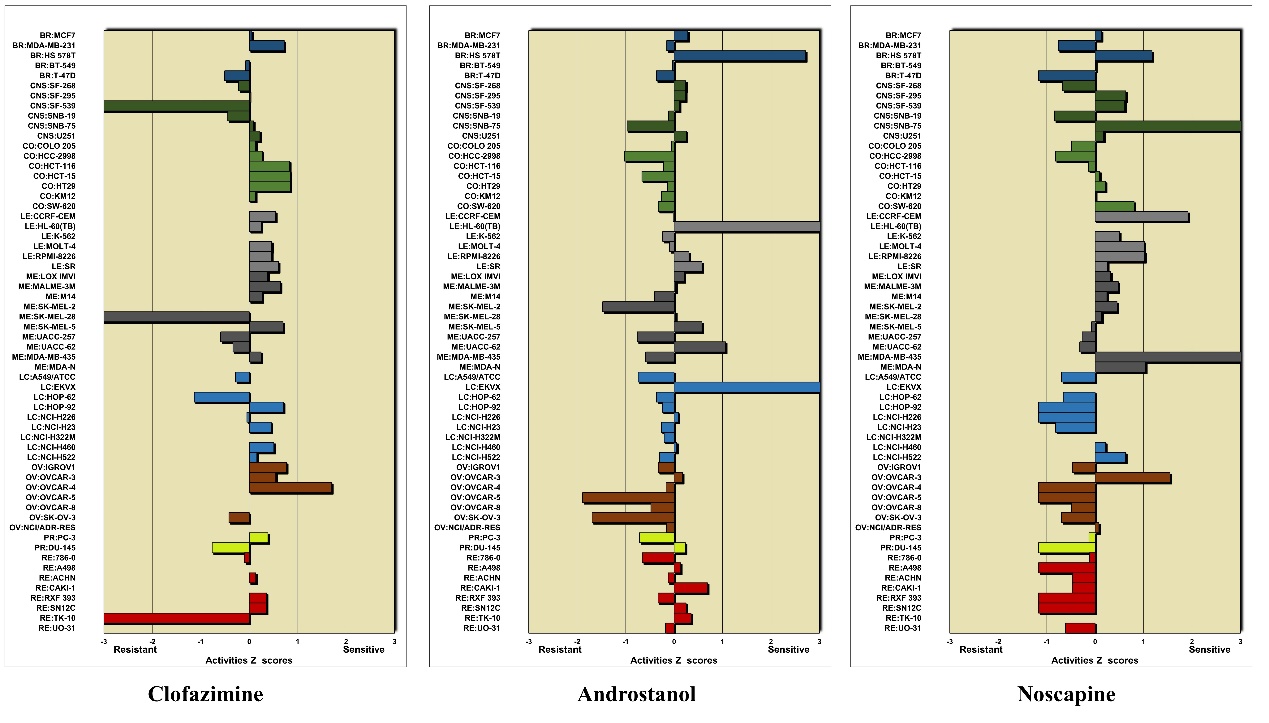


Figure S5. Drug activities Z-scores among different CRC cell lines calculated by online tool CellMiner.


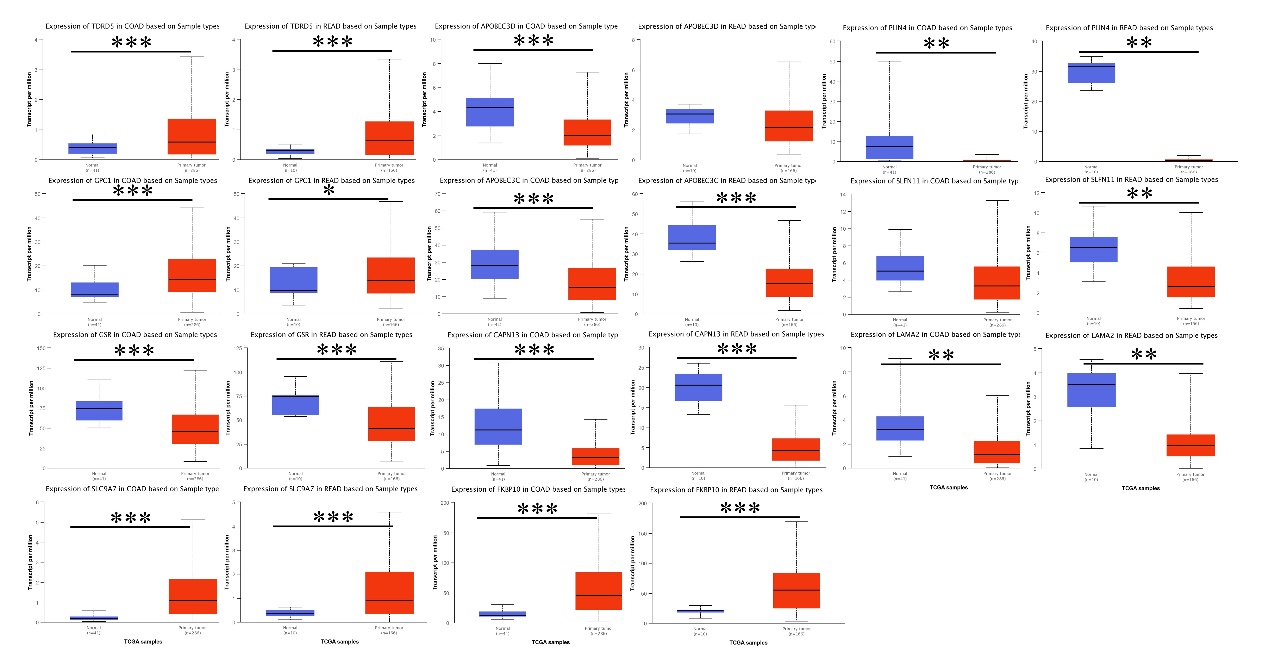


Figure S6. mRNA expression levels of the 12 genes validated using UALCAN online tool. (Data of ERFE are not available in this database)
